# Interactions between B cells and T follicular regulatory cells enhance susceptibility to *Brucella* infection independent of the anti-*Brucella* humoral response

**DOI:** 10.1371/journal.ppat.1011672

**Published:** 2023-09-18

**Authors:** Alexis S. Dadelahi, Mostafa F. N. Abushahba, Bárbara Ponzilacqua-Silva, Catherine A. Chambers, Charles R. Moley, Carolyn A. Lacey, Alexander L. Dent, Jerod A. Skyberg

**Affiliations:** 1 Department of Veterinary Pathobiology, College of Veterinary Medicine, University of Missouri, Columbia, Missouri, United States of America; 2 Laboratory for Infectious Disease Research, University of Missouri, Columbia, Missouri, United States of America; 3 Department of Zoonoses, Faculty of Veterinary Medicine, Assiut University, Assiut, Egypt; 4 Department of Microbiology and Immunology, Indiana University School of Medicine, Indianapolis, Indiana; University of California, Davis, UNITED STATES

## Abstract

Brucellosis, caused by facultative, intracellular *Brucella* spp., often results in chronic and/or lifelong infection. Therefore, *Brucella* must employ mechanisms to subvert adaptive immunity to cause chronic infection. B lymphocytes enhance susceptibility to infection with *Brucella* spp. though the mechanisms remain unclear. Here we investigated the role of antibody secretion, B cell receptor (BCR) specificity, and B cell antigen presentation on susceptibility to *B*. *melitensis*. We report that mice unable to secrete antibody do not display altered resistance to *Brucella*. However, animals with B cells that are unable to recognize *Brucella* through their BCR are resistant to infection. In addition, B cell MHCII expression enhances susceptibility to infection in a CD4^+^ T cell-dependent manner, and we found that follicular B cells are sufficient to inhibit CD4^+^ T cell-mediated immunity against *Brucella*. B cells promote development of T follicular helper (T_FH_) and T follicular regulatory (T_FR_) cells during *Brucella* infection. Inhibition of B cell and CD4^+^ T cell interaction via CD40L blockade enhances resistance to *Brucella* in a B cell dependent manner concomitant with suppression of T_FH_ and T_FR_ differentiation. Conversely, PD-1 blockade increases *Brucella* burdens in a B and CD4^+^ T cell dependent manner while augmenting T regulatory (T_Reg_) and T_FR_ responses. Intriguingly, T_FR_ deficiency enhances resistance to *Brucella* via a B cell dependent, but antibody independent mechanism. Collectively, these results demonstrate B cells support T_FR_ responses that promote susceptibility to *Brucella* infection independent of the antibody response.

## Author summary

*Brucella* can cause a life-long infection in humans, mice and livestock making it an ideal organism for investigating the mechanisms that pathogens employ to subvert host immunity and cause chronic infection. Here, we determined how B cell effector functions enhance susceptibility to *Brucella*. We found that animals with B cells that are unable to recognize *Brucella* through their BCR are resistant to infection. We also found that B cells alter the function of CD4^+^ T cells during *Brucella* infection in a MHCII- and CD40:CD40L-dependent manner. Moreover, we show that B cells promote development of T follicular regulatory cells that in turn enhance susceptibility to *Brucella* in an antibody independent manner. This was of particular interest, because to our knowledge, an antibody independent function of T follicular regulatory cells in modulating susceptibility to infection has not been previously reported. Collectively, this study highlights mechanisms by which *Brucella* infection subverts B and CD4^+^ T cell interactions to promote host susceptibility to infection.

## Introduction

Consistently ranked by the World Health Organization as one of the world’s most common neglected zoonoses, brucellosis remains a major impediment to human health and economic stability [1,2]. Due to non-descript clinical signs and inadequate testing procedures, it is likely that global brucellosis incidence is grossly underestimated, and the chronicity of infection further complicates measures to mitigate disease [3,4]. Infection occurs frequently following consumption of unpasteurized dairy products or contact with tissues from livestock infected with the gram negative, facultative intracellular bacteria, *Brucella* [5–7]. Chronic disease is a frequent outcome of infection, even when aggressive antibiotic therapy is employed, and is typified by onset of various sequelae including relapsing undulant fever, arthritis, and neurobrucellosis [2,5]. Currently, our understanding of mechanisms that allow *Brucella* spp. to circumvent protective host responses are lacking and represent a major limitation for rational vaccine and therapeutic development.

IFN-γ is crucial to effective control of infection [8,9], and endogenous Th1, Th17 and CD8^+^ T cell responses, in the right context, can confer some level of protection [2,10–15]. However, *Brucella* can cause a lifelong infection in humans, livestock, and mice [10,16,17], and robust protection (that which results in one log or greater reduction in bacterial burden) typically fails to arise prior to one-month post infection, underscoring the inefficiency of this response.

B cell deficient mice display enhanced resistance to *Brucella* that is not altered by passive transfer of antibody [10,18]. We previously reported B cells require CD4^+^ T cells to promote susceptibility to infection, indicating that CD4^+^ T cell and B cell interactions are detrimental to control of *Brucella* [19]. However, the nature of this interaction remains undefined. T_FH_ comprise a subset of CD4^+^ T cells specialized in providing essential help to follicular B cells (Fo B) during the germinal center (GC) response via CD40L, IL-21 and IL-4 [20]. Efficient class switching, affinity maturation, and generation of memory and plasma B cells during infection depend upon this crucial interaction; however, tight regulation of this process is vital for prevention of self-reactivity while also facilitating control of infection [20]. The role of T_FR_ in fine-tuning this response has recently come to light. T_FR_ share many characteristics in common with T_FH_ populations including expression of Bcl6, CXCR5, ICOS, and PD-1 [21]. Additionally, T_FR_ share various characteristics with T_Reg_ including expression of FoxP3, GITR, and CTLA-4 [22–25]. Compelling evidence points to a regulatory role for T_FR_ throughout the GC response, which includes conditioning both the magnitude and quality of the response via suppression of T_FH_ and germinal center B cells (GC B) [22–24,26–33]. While this function is likely dependent on the enhanced ability of T_FR_ to suppress B cell responses specifically [22,24,26,34], the mechanisms involved in this process are largely undefined.

Here we show both B cell receptor (BCR) specificity and B cell antigen presentation function to enhance susceptibility to *Brucella*. Fo B are sufficient for inhibition of protective CD4^+^ T cell responses, and B cells promote T_Reg_ and T_FR_ populations which were associated with enhanced susceptibility to *Brucella*. Using T_FR_ deficient mice, we demonstrate T_FR_ enhance susceptibility to *Brucella* in a B cell-dependent, but antibody independent, manner.

## Results

### Role of BCR specificity and secreted antibody in uptake of *Brucella* by B cells and host susceptibility to infection

**Fig 1:**
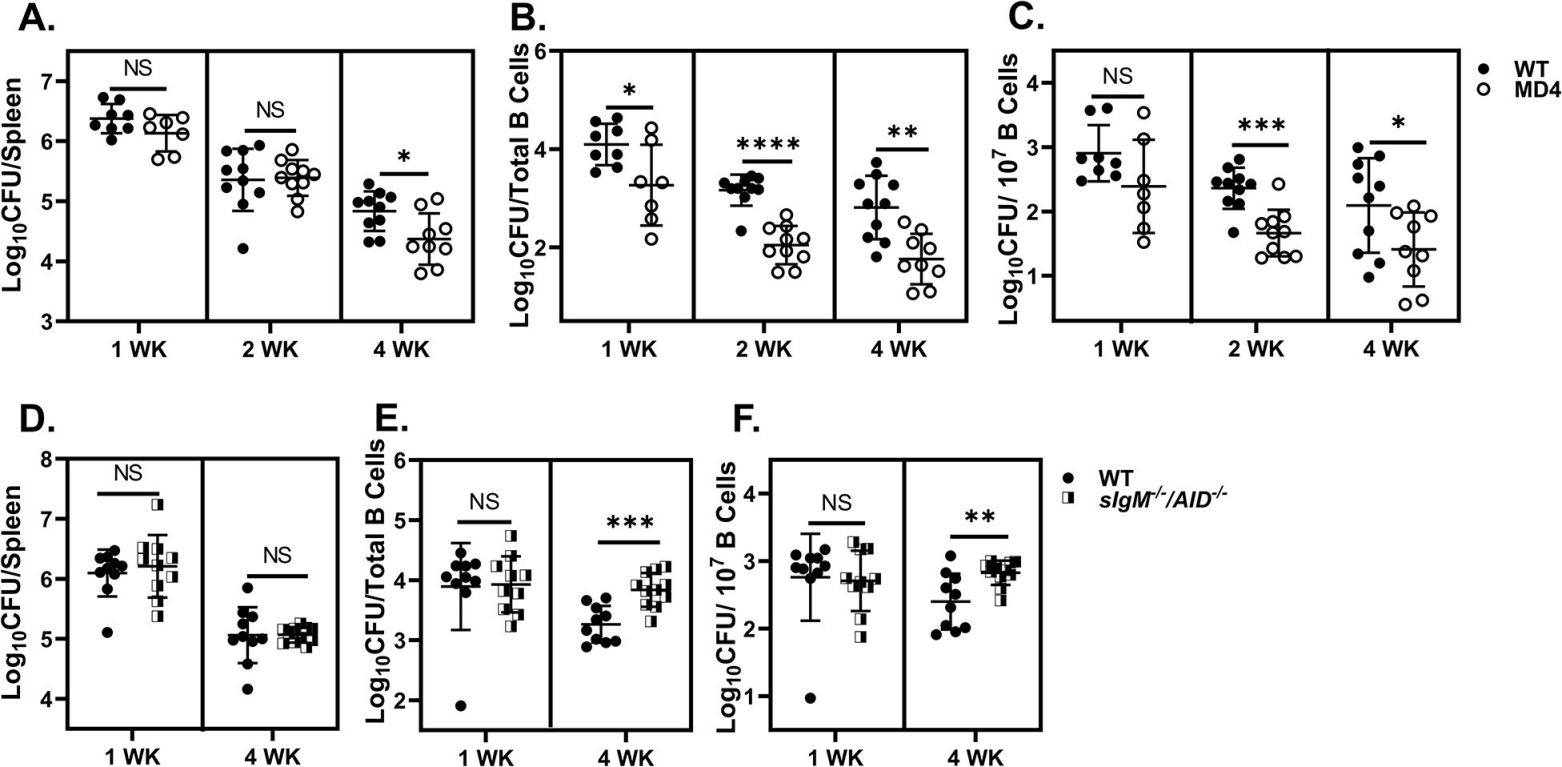
Role of BCR specificity and secreted antibody in susceptibility to infection and B cell uptake of *Brucella*. MD4 mice or WT littermates (n = 7-10/group/timepoint) were challenged i.p. with 1x10^5^ CFUs of *B*. *melitensis* 16M. **(A)** Splenic bacterial burdens measured at one-, two-, and four-weeks post infection. **(B)** Viable intracellular *Brucella* recovered from total sorted CD19^+^ splenic B cells at one, two- and four-weeks post infection. **(C)** Viable intracellular *Brucella* burden per sorted B cell recovered at one, two- and four weeks post challenge. **(D)** Splenic bacterial loads of WT and *sIgM*^-/-^/*AID*^-/-^ mice (n = 10-12/group/timepoint) at one- and four-weeks post challenge with *B*. *melitensis*. **(E)** Total viable intracellular *Brucella* recovered from sorted CD19^+^ splenic B cells at one- and four-weeks post infection in WT and *sIgM*^-/-^/*AID*^-/-^ animals. **(F)** Viable intracellular *Brucella* burden per sorted B cell recovered at one-, and four-weeks post challenge in WT and *sIgM*^-/-^/*AID*^-/-^ mice. **(A-F)** Data are combined from 2 independent experiments per time point.

BCR-mediated antigen uptake is 100- to 1000-fold more efficient for cognate T cell activation via MHCII than BCR-independent routes of B cell antigen presentation [35]. To investigate whether BCR specificity for *Brucella* alters susceptibility to infection, we challenged WT and MD4 mice, in which ~90% of B cells express a BCR specific for the irrelevant antigen hen egg lysozyme (HEL) [36] with *B*. *melitensis*. Significantly fewer *Brucella* were recovered from spleens of MD4 mice by four weeks post challenge (Fig 1A). Levels of total IgG and IgM were reduced ~10-fold in MD4 mice (S1A Fig), similar to what has been observed in MD4 mice in other infection models [37]. However, MD4 animals generated ~1000-fold less anti-*Brucella* IgG compared to WT controls at both two- and four-weeks post infection (S1B and S1C Fig), confirming a reduced affinity for *Brucella* antigen by MD4 B cells. Taken together, these results suggest that BCR specificity for *Brucella* promotes susceptibility to infection.

Because B cell uptake of antigen is more efficient via the BCR than alternate mechanisms [35], we investigated the impact of BCR specificity on *Brucella* uptake by B lymphocytes. Compared to WT controls, B cell lysates collected from MD4 mice consistently harbored fewer *Brucella* with ~10-fold reduction at one-, two-, and four-weeks post challenge (Fig 1B). MD4 and WT mice harbor similar levels of splenic *B*. *melitensis* through the first two weeks of infection (Fig 1A) indicating that reduced B cell uptake of *Brucella* is a B cell specific effect rather than a result of diminished total bacterial burden in MD4 animals. Because we recovered fewer B cells from MD4 animals relative to WT mice (S1D Fig), we calculated the number of *B*. *melitensis* CFUs recovered per B cell and found this was also significantly reduced at two- and four-weeks post infection in MD4 animals (Fig 1C). Collectively, these data indicate that a diminished capacity for BCR-mediated recognition of *Brucella* impairs uptake of *Brucella* by B cells, which in turn may alter host susceptibility to infection.

Opsonization of *Brucella* with IgM from previously infected mice enhances uptake by B cells *in vitro* [4]. To investigate the effect of *Brucella*-specific antibody on uptake *in vivo* we employed *sIgM*^*-/-*^*/AID*^*-/-*^ mice which express a polyclonal BCR but do not secrete IgM nor generate class-switched antibodies [38]. Total splenic *Brucella* burdens were similar among WT and *sIgM*^*-/-*^*/AID*^*-/-*^ animals at both one- and four-weeks post-infection (Fig 1D). In contrast to MD4 mice (Fig 1B), *sIgM*^*-/-*^*/AID*^*-/-*^ mice displayed similar intracellular *Brucella* B cell burdens one week post infection and increased B cell burdens at four weeks post infection relative to WT mice (Fig 1E and 1F). These data indicate that secreted antibody does not alter susceptibility to infection, nor is it absolutely required for *Brucella* entry into B cells.

### B cell antigen presentation promotes deleterious CD4^+^ T cell responses

**Fig 2:**
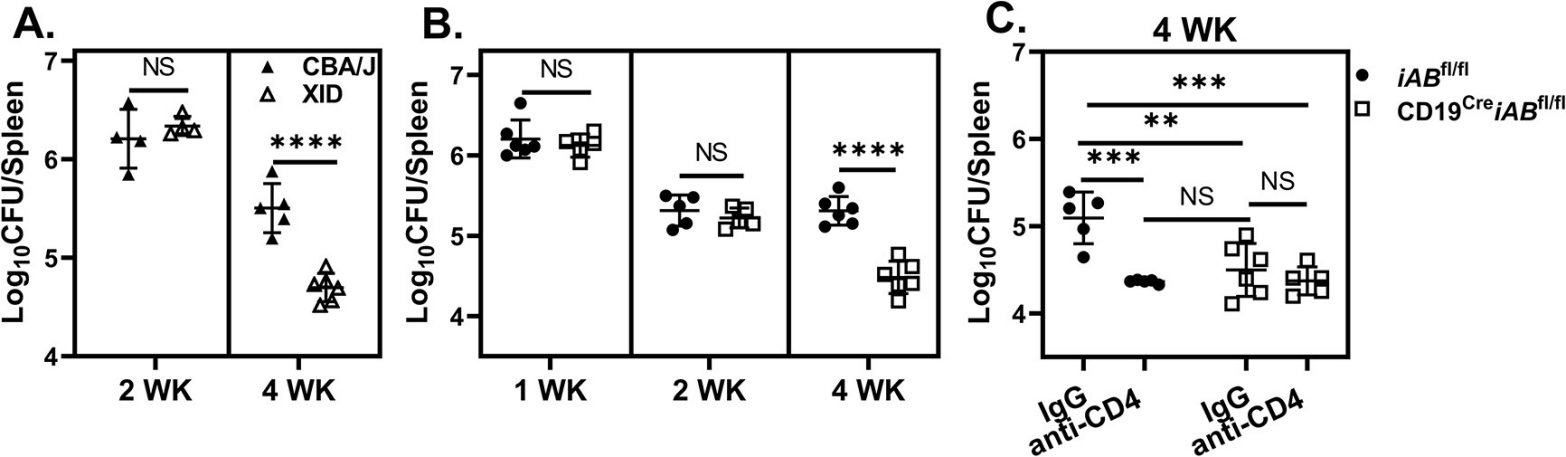
B cell antigen presentation promotes deleterious CD4^+^ T cell responses during *Brucella* infection. **(A)** Splenic *Brucella* loads of XID and CBA/J animals (n = 4-6/group/timepoint) following challenge with *B*. *melitensis* at two- and four-weeks post infection. **(B)** Splenic *Brucella* burden of CD19^Cre^*iAB*^fl/fl^ or *iAB*^fl/fl^ littermates (n = 3-7/group/timepoint) at one-, two-, and four-weeks post infection. **(C)** CD19^Cre^*iAB*^fl/fl^ and *iAB*^fl/fl^ mice were treated with CD4^+^ T cell-depleting antibody, or IgG isotype, and challenged with *B*. *melitensis*. Four weeks post infection splenic *Brucella* burdens were compared. Data are representative of at least two independent experiments.

BCR-mediated antigen uptake, trafficking and presentation are regulated by Bruton’s tyrosine kinase (Btk) [39]. Therefore, we infected mice with a mutational defect in Btk (XID) and compared control of infection to CBA/J control mice. Bacterial burdens were similar two weeks post infection, but XID mice displayed enhanced resistance by four weeks post challenge (Fig 2A) suggesting Btk-dependent B cell antigen presentation enhances susceptibility to brucellosis. In addition to disrupting BCR-mediated antigen presentation, Btk dysfunction suppresses B-1a cell development in XID mice [40] which can impact control of infection [41]. However adoptive transfer of B-1a cells from naive CBA/J donors to XID mice prior to challenge did not alter control of *Brucella* (S1E Fig), indicating resistance in XID animals is independent of B-1a cell deficiency.

To further examine the role of B cell antigen presentation on CD4^+^ T cell responses to *Brucella*, we compared control of infection in B cell specific MHCII deficient mice (CD19^Cre^*iAB*^fl/fl^) and *iAB*^fl/fl^ control animals and found B cell specific MHCII deficiency enhanced resistance to *Brucella* four weeks post infection (Fig 2B). Additionally, while depletion of CD4^+^ T cells from *iAB*^fl/fl^ mice enhanced resistance to infection, CD4^+^ T cell depletion had no effect in CD19^Cre^*iAB*^fl/fl^ mice at four weeks post infection (Fig 2C). This indicated that B cell MHCII expression promotes CD4^+^ T cell responses that enhance susceptibility to *Brucella*.

### Follicular B cells Promote Susceptibility to *Brucella*

**Fig 3:**
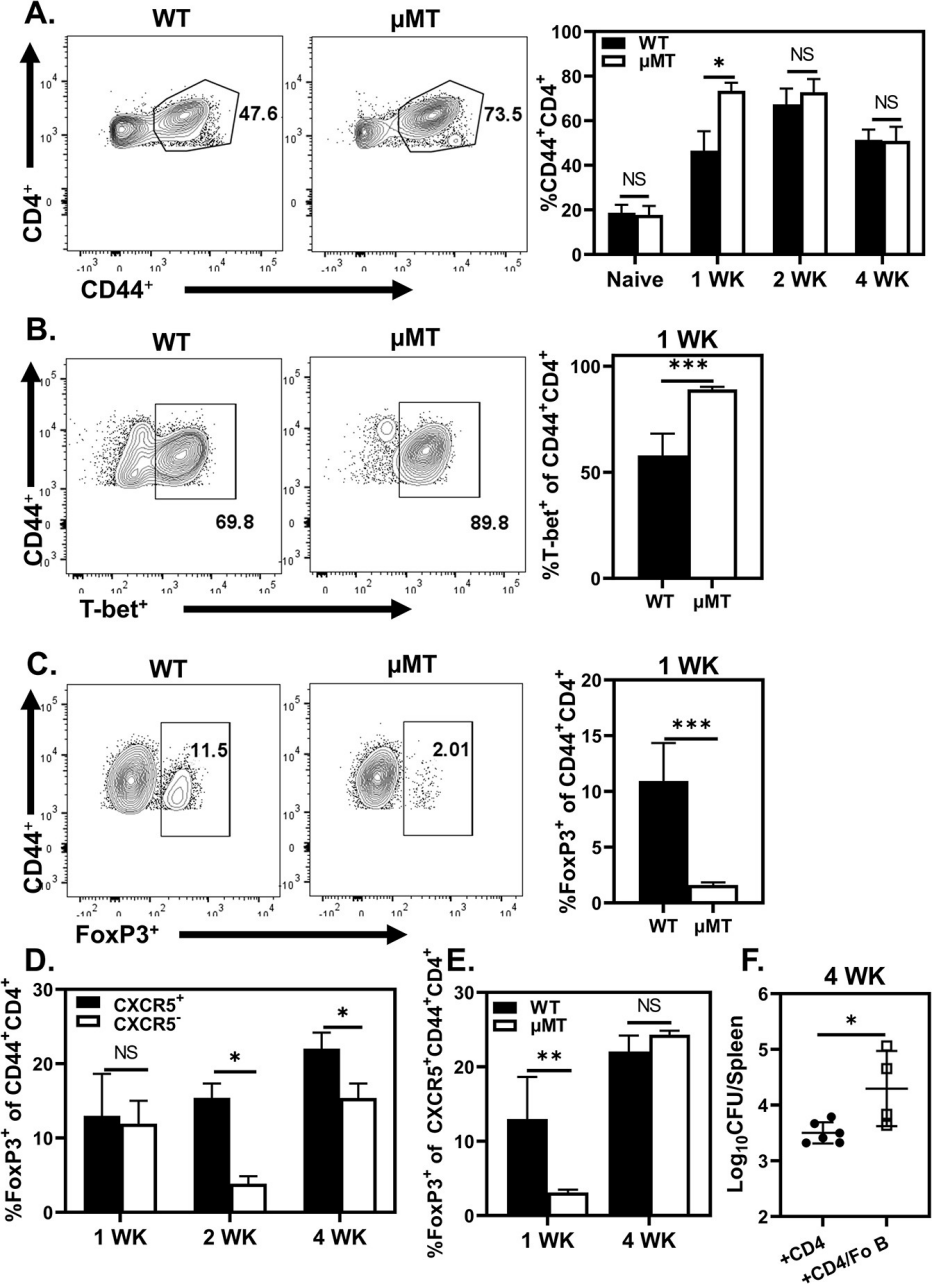
Fo B inhibit CD4^+^ T cell immunity to *Brucella*. Splenic CD4^+^ T cell responses assessed via flow cytometry in naïve (n = 2) or *B*. *melitensis* infected WT and μMT mice (n = 3-6/group/time point) at one-, or four-weeks post infection. Quantification of CD44 expression on CD4^+^ T cells **(A)** along with T-bet **(B)**, and FoxP3 **(C)** expression on CD44 expressing CD4^+^ T cells**. (D)** Proportion of FoxP3^+^ cells among CXCR5^+^ and CXCR5^-^ CD44^+^CD4^+^ T cells at one-, two-, and four-weeks post infection in WT animals. **(E)** Proportion of FoxP3^+^ cells amongst CXCR5^+^CD44^+^CD4^+^ T cells in WT and μMT mice one- and four-weeks post infection. **(F)** *Rag1*^-/-^ mice (n = 3-7/treatment) adoptively transferred either CD4^+^ T cells alone, or CD4^+^ T cells in combination with purified Fo B cells one day prior to *B*. *melitensis* challenge. Data are representative of at least two independent experiments.

We next compared CD4^+^ T cell phenotypes in B cell deficient (μMT) and WT mice infected with *B*. *melitensis*. At one week post infection, we observed a significant increase in the percentage of activated (CD44^+^) CD4^+^ T cells in μMT mice (Fig 3A). μMT mice also displayed ~30% increase in T-bet expression on CD44^+^CD4^+^ T cells one week post infection, though this difference dissipated two weeks post challenge (Figs 3B and S1F). FoxP3 expression on CD44^+^CD4^+^ T cells was significantly decreased among μMT animals compared to WT at one- and two weeks post infection (Figs 3C and S1G), suggesting B cells may drive T_Reg_ differentiation in response to *Brucella*. Because μMT mice can exhibit altered T cell development [42], we confirmed our findings with adoptive transfer experiments. *Rag1*^*-/-*^ animals received CD4^+^ T cells alone, or both CD4^+^ T and B cells from WT donors prior to challenge with *B*. *melitensis*. Similar to our findings in μMT mice, by two weeks post infection co-transfer of B cells enhanced FoxP3 expression and diminished T-bet expression on CD44^+^CD4^+^ T cells (S1H and S1I Fig).

Further analysis of CD44^+^CD4^+^ T cells in WT animals revealed FoxP3 expression was preferentially enhanced among CXCR5^+^ populations vs CXCR5^-^ populations (15.40% ±1.93% vs 3.6 ±1.22) at two- and four-weeks post infection (Fig 3D). WT mice also display increased FoxP3^+^ cell frequencies amongst CXCR5^+^CD44^+^CD4^+^ T cell populations one week post infection compared to μMT animals (Fig 3E), suggesting B cells promote T_Reg_ development amongst CXCR5^+^CD44^+^CD4^+^ T cells during *Brucella* infection.

CXCR5 expression facilitates CD4^+^ T cell trafficking to B cell follicles and subsequent interaction with cognate Fo B [43–45]. To test the role of Fo B during *Brucella* infection, we adoptively transferred CD4^+^ T cells alone, or both CD4^+^ T cells and Fo B into *Rag1*^*-/-*^ mice prior to challenge. At four weeks post infection, co-transfer of Fo B with CD4^+^ T cells resulted in ~10-fold increase in splenic *Brucella* loads compared to animals transferred CD4^+^ T cells alone (Fig 3F), demonstrating Fo B inhibit CD4^+^ T cell responses following *B*. *melitensis* infection. Notably, we observed no marked effect in adoptive transfer experiments in which B-1a cells were co-transferred with CD4^+^ T cells (S1J Fig).

### CD40L blockade enhances resistance to *Brucella*

**Fig 4:**
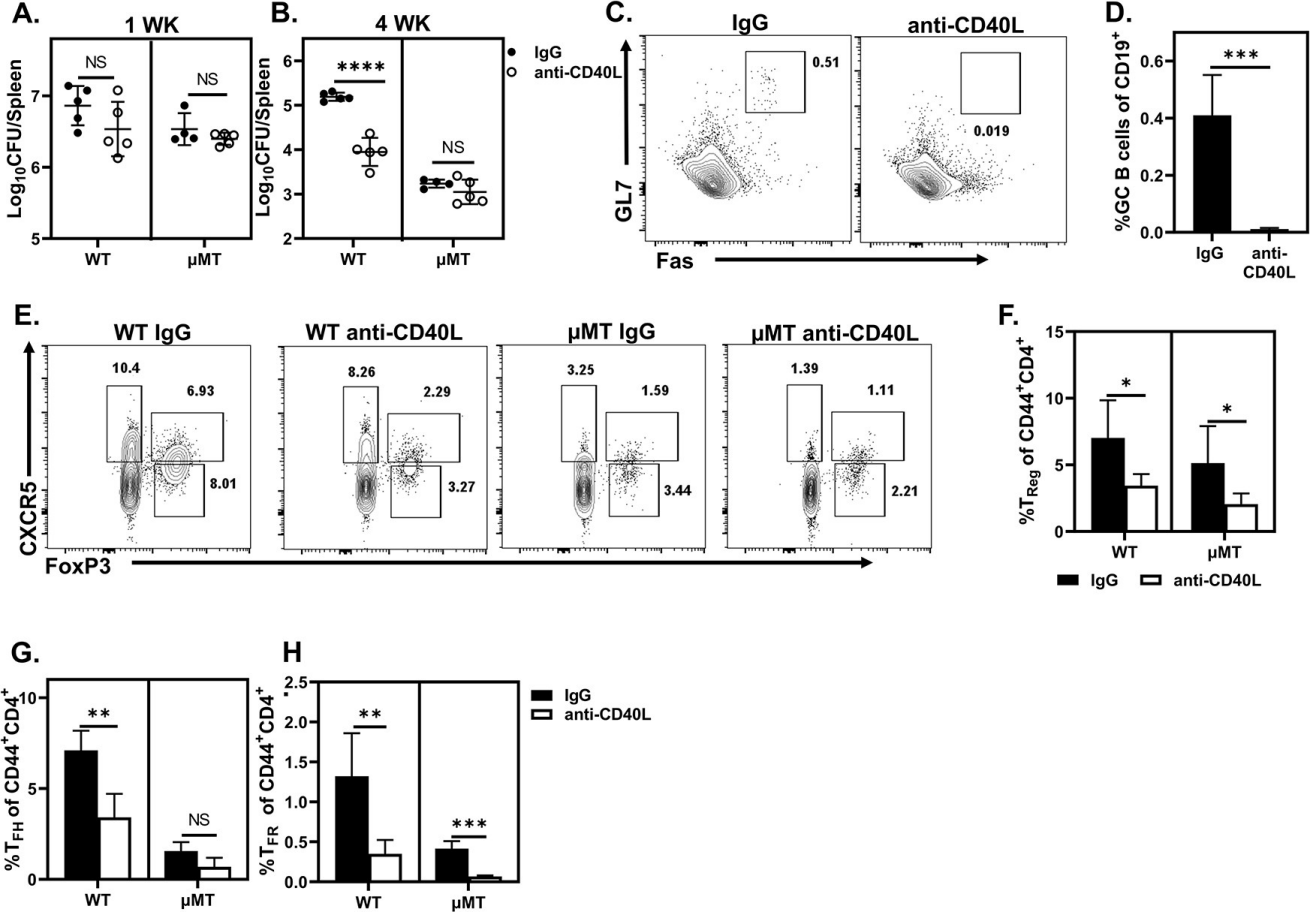
CD40L blockade enhances resistance to *Brucella*. **(A-H)** Mice (n = 3-5/group) were treated with CD40L blocking antibody or IgG. **(A-B)** Splenic *Brucella* burdens were measured in WT and μMT mice one **(A)** and four **(B)** weeks post infection. Representative plots **(C)** and quantification **(D)** of the percentage of GC B cells (CD19^+^CD43^-^GL7^+^Fas^+^) among CD19^+^ B cells in isotype or anti-CD40L treated WT animals. **(E-G)** Representative plots showing CXCR5 and FoxP3 expression **(E)** and quantification **(F-H)** of the percentage of T_Reg_ (FoxP3^+^CXCR5^-^) **(F)**, T_FH_ (FoxP3^-^ICOS^+^CXCR5^+^) **(G)**, and T_FR_ (FoxP3^+^ICOS^+^CXCR5^+^) **(H)** amongst CD44^+^CD4^+^ T cells in IgG and anti-CD40L-treated WT and μMT mice four weeks post infection. Data are representative of at least two independent experiments.

CD40:CD40L interactions between T_FH_, and Fo B following priming are requisite for generation of GC responses [46,47]. Thus, we hypothesized inhibition of CD40:CD40L interactions could suppress the deleterious effects resulting from interaction of CD4^+^ T cells and Fo B during *Brucella* infection. Interestingly, CD40L blockade in WT, but not uMT animals, enhanced control of splenic *Brucella* burdens four weeks post infection (Fig 4A and 4B), indicating the deleterious effect of CD40:CD40L interactions is B cell dependent. CD40L blockade also suppressed GC B cell responses (Figs 4C, 4D, S2A and S2B) and the proportion of T_Reg_, T_FH_, and T_FR_ four-weeks post challenge (Figs 4E–4H and S2C–S2E). Collectively these data establish that interruption of CD40:CD40L interactions enhances resistance to *Brucella*, and suggest disrupting GC B, T_Reg_, T_FH_, and/or T_FR_ populations may improve control of infection.

### Bcl6 expression in CD4 T cells protects the host against *Brucella*

**Fig 5:**
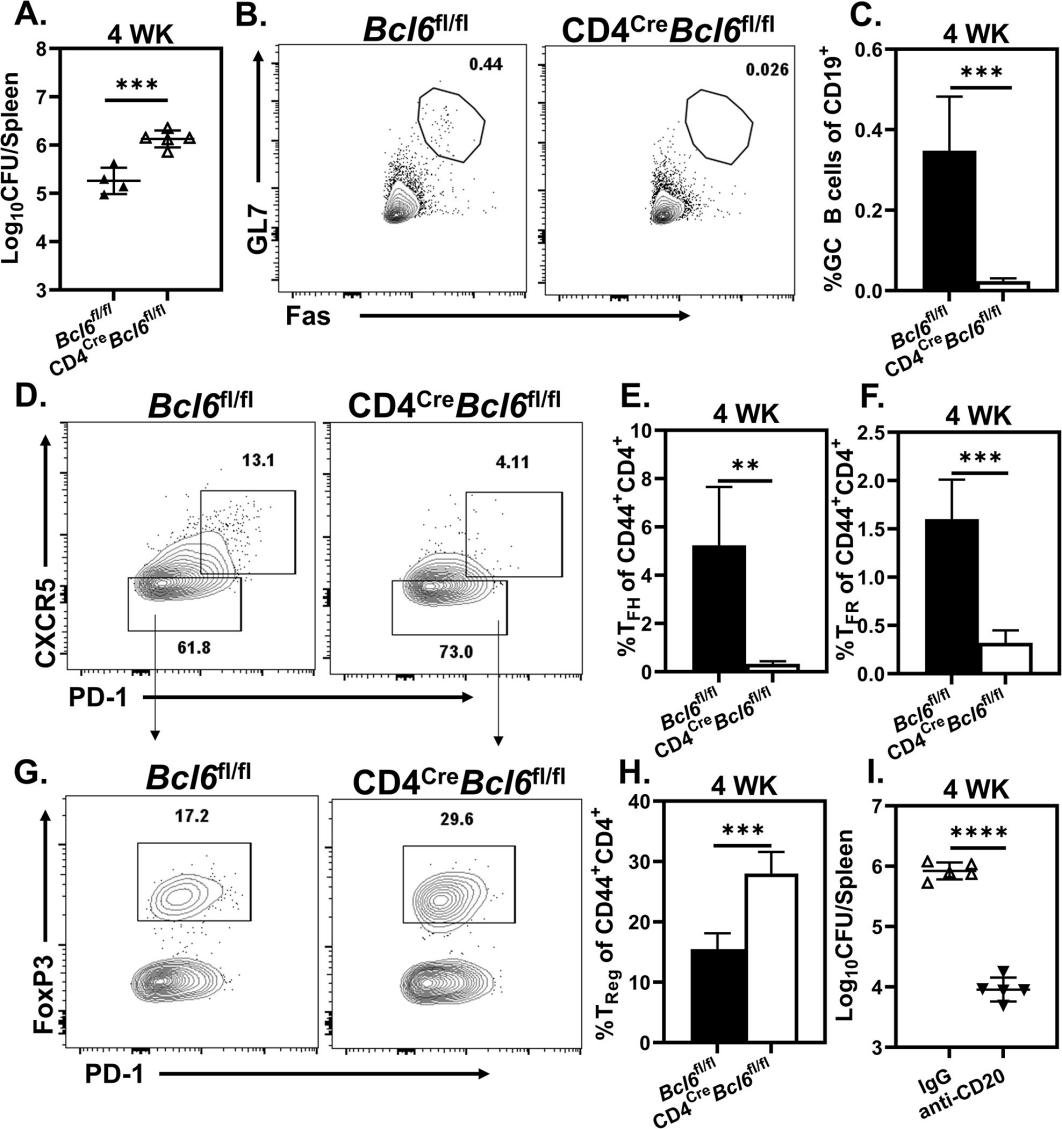
Lack of Bcl6 expressing CD4^+^ T cells impairs protection against *Brucella*. *Bcl6*^fl/fl^ or CD4^Cre^*Bcl6*^fl/fl^ mice (n = 4-5/group) were challenged with *B*. *melitensis*. **(A)** Splenic bacterial burdens measured four weeks post infection. **(B-C)** Representative plots **(B)** and quantification **(C)** of the percentage of GC B cells amongst CD19^+^ cells four weeks post infection. **(D-F)** Representative plots showing CXCR5 and PD-1 expression **(D)** and quantification of the percent of T_FH_ (FoxP3^-^PD-1^+^CXCR5^+^) **(E)** and T_FR_ (FoxP3^+^PD-1^+^CXCR5^+^) **(F)** amongst CD44 expressing CD4^+^ T cells four weeks post infection. **(G-H)** Representative plots showing FoxP3 and PD-1 expression **(G)** and quantification **(H)** of the percentage of T_Reg_ (FoxP3^+^CXCR5^-^) among CD44 expressing CD4^+^ T cells four weeks post infection. **(I)** Splenic bacterial burdens of CD4^Cre^*Bcl6*^fl/fl^ animals (n = 5/treatment) treated with anti-CD20 or IgG four weeks post *Brucella* challenge. Data are representative of at least two independent experiments.

Expression of Bcl6 in CD4^+^ T cells is essential to differentiation of both T_FH_ and T_FR_ [22,48,49]. To ascertain whether *Brucella* infection is exacerbated by B cell interaction with Bcl6 expressing CD4^+^ T cells, we infected CD4^Cre^*Bcl6*^fl/fl^ mice with *B*. *melitensis*. While depletion of total CD4^+^ T cells enhances resistance (Fig 2C), deletion of Bcl6^+^CD4^+^ T cells increased susceptibility to *Brucella* four weeks post challenge (Fig 5A). Thymocytes express both CD8 and CD4 during early development, and Bcl6 is expressed by some splenic CD8^+^ T cells [50,51]. However, we found enhanced susceptibility in CD4^Cre^*Bcl6*^fl/fl^ mice did not require CD8^+^ T cells (S2F Fig). Similar to previous reports [25,52], deletion of Bcl6 in CD4^+^ T cells suppressed differentiation of GC B cells, T_FH_, and T_FR_, though T-bet expression remained similar between groups (Figs 5B–5F and S2G). While CD4^Cre^*Bcl6*^fl/fl^ mice had markedly reduced levels of GC B cells (Fig 5B and 5C), B cell depleted CD4^Cre^*Bcl6*^fl/fl^ animals displayed ~100-fold decrease in splenic *Brucella* loads compared to controls (Fig 5I) suggesting GC B cells are not essential for B cell mediated susceptibility to *Brucella*. We also observed a marked increase in both the frequency and absolute number of T_Reg_ following *Brucella* infection in CD4^Cre^*Bcl6*^fl/fl^ animals (Figs 5G, 5H and S2H). This was of interest given that CD40L blockade, which results in protection associated with suppression of T_FH_, T_FR_ and GC B, significantly reduced T_Reg_ frequencies (Fig 4F–4H). Taken together, these findings suggest one or more Bcl6 expressing CD4^+^ T cell populations may be necessary for efficient control of infection, or that outgrowth of T_Reg_ in CD4^Cre^*Bcl6*^fl/fl^ mice drives enhanced susceptibility to challenge.

### PD-1 blockade enhances susceptibility to *Brucella*

**Fig 6:**
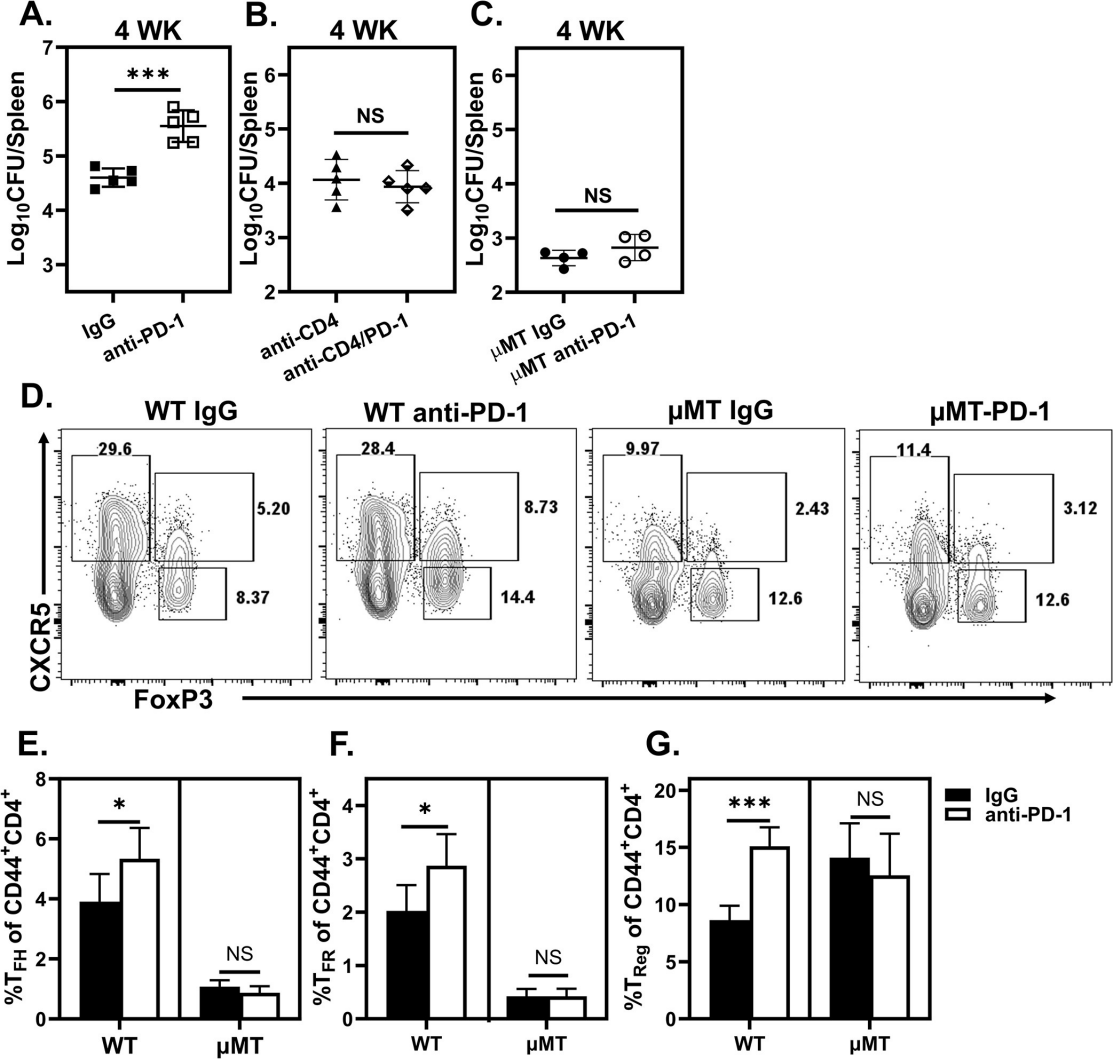
PD-1 blockade enhances susceptibility to *Brucella*. WT and/or μMT mice (n = 4-5/treatment) were treated with PD-1 blocking antibody, CD4^+^ T cell depleting antibody, PD-1 blocking and CD4-depleting antibody, or isotype control. **(A & C)** Splenic bacterial loads in IgG and PD-1 blocked WT **(A)** or μMT **(C)** mice. **(B)** Splenic bacterial burden of CD4-depleted and CD4-depleted/PD-1 blocked WT mice. **(D)** Representative flow plots showing CXCR5 and FoxP3 expression and the percentage of T_FH_ (FoxP3^-^ICOS^+^CXCR5^+^) **(E)**, T_FR_ (FoxP3^+^ICOS^+^CXCR5^+^) **(F)**, and T_Reg_ (FoxP3^+^CXCR5^-^) **(G)** present in IgG and anti-PD-1 treated WT and μMT mice spleens four weeks post challenge. **(E-G)** Quantification of the percentage of each indicated population of CD44 expressing CD4^+^ T cells four weeks post challenge. Data are representative of at least two independent experiments.

CD40L blockade and Bcl6^+^CD4^+^ T cell deficiency both dampen T_FH_ responses yet have opposing effects on control of infection (Figs 4 and 5). Combined with our finding that B cells promote FoxP3 expression amongst CXCR5^+^CD44^+^CD4^+^ T cells, we questioned whether altering T_Reg_ and T_FR_ frequencies would alter control of *Brucella*. PD-1 regulates both T_Reg_ and T_FR_ differentiation and function [27,53,54]. Intriguingly, anti-PD-1 treated WT animals exhibited significantly increased bacterial loads compared to controls four weeks post *Brucella* challenge (Fig 6A). While PD-1 expression is a key trait of T_FH_ [27,55,56], various cell populations signal through PD-1 [57]. However, neither PD-1 blocked CD4^+^ T cell-depleted mice nor μMT animals had exacerbated infection (Fig 6B and 6C), demonstrating this effect is both CD4^+^T and B cell dependent. PD-1 blockade enhanced T_FH_, T_Reg_ and T_FR_ proportions during infection in WT animals, while these populations remained unchanged in anti-PD-1 treated μMT animals (Fig 6D–6G). Therefore, one or more of these populations may drive B cell-dependent susceptibility to *Brucella* infection.

### B cells are not absolutely required for T_Reg_- mediated susceptibility to *Brucella*

As PD-1 blockade enhances susceptibility to infection as well as T_Reg_ and T_FR_ outgrowth (Fig 6A, 6F and 6G), we investigated the contribution of T_Reg_ to control of infection. DEREG mice bear a diphtheria toxin receptor transgene under the control of the Foxp3 promoter, allowing for depletion of FoxP3 expressing cells by administration of diphtheria toxin (DTX). Because efficient FoxP3^+^ cell depletion is short-lived [58,59], we treated DEREG and WT groups with (DTX) on days 14 and 15 post challenge and confirmed FoxP3 expressing CD4^+^ T cells were diminished (S3A Fig). In line with previous reports suggesting T_Reg_ cells inhibit control of brucellosis [60,61], T_Reg_ depletion significantly enhanced resistance to *Brucella* at four weeks post-infection (S3B Fig). DTX-treated DEREG mice also presented with a decreased frequency of B cells compared to DTX-treated WT animals (S3C and S3D Fig); however, GC B and T_FH_ proportions were increased in DTX treated DEREG animals compared to WT controls (S3E, S3F,S3I and S3K Fig). T-bet expressing CD4^+^ T effector levels were similar among FoxP3-depleted and WT groups (S3G Fig). While B cells enhance the proportion of T_Reg_ during *Brucella* infection (Figs 3C, 3E, S1G and S1H), depletion of T_Reg_ in both control and B cell depleted mice enhanced resistance to infection (S3B and S3H Fig) indicating the deleterious effect of T_Reg_ is not entirely B cell dependent.

### T_FR_ enhance susceptibility to *Brucella* infection in a B cell dependent, but antibody independent, manner

**Fig 7:**
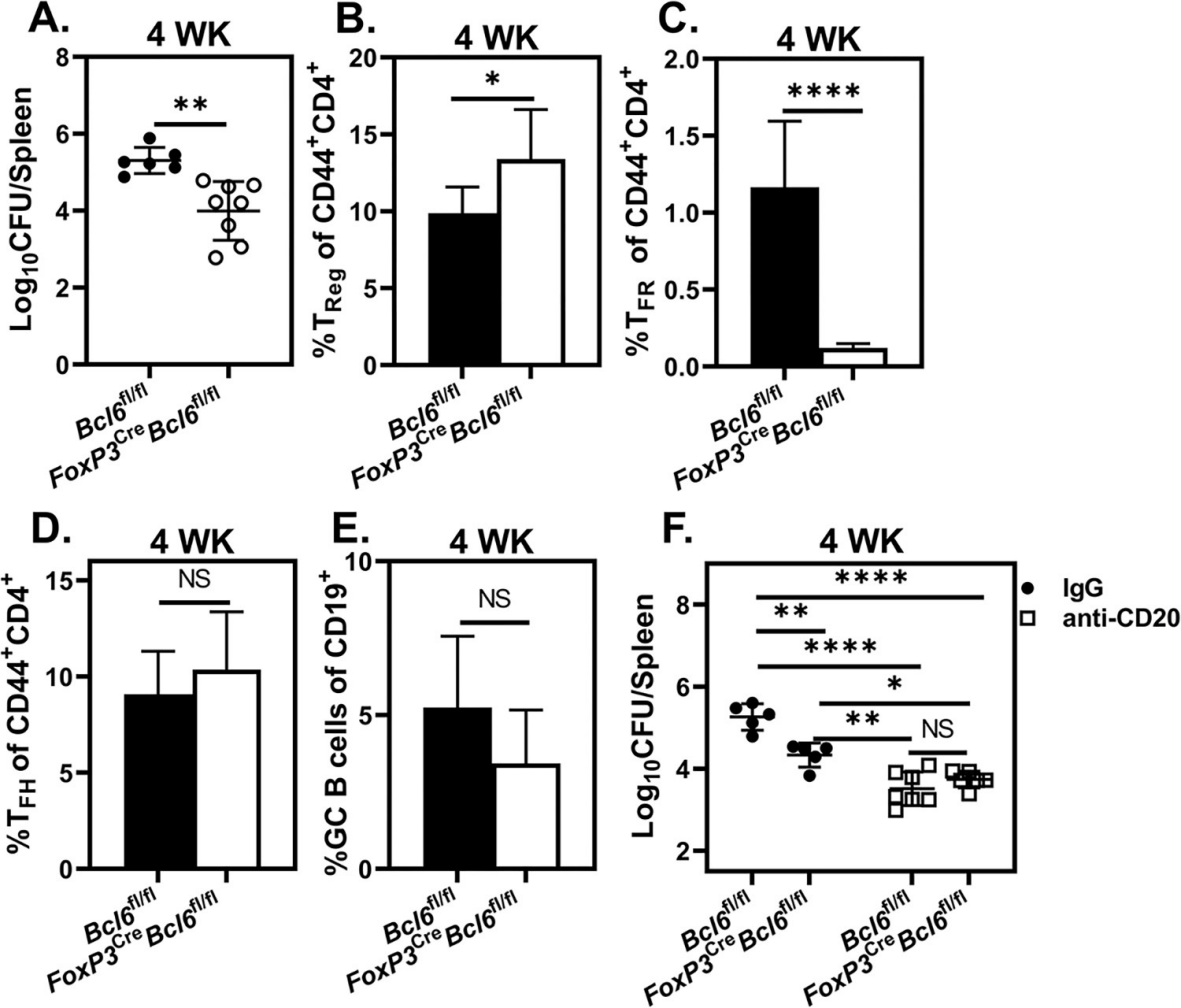
T_FR_ enhance susceptibility to *Brucella*. **(A)** Splenic *Brucella* burdens of *Bcl6*^fl/fl^ and *FoxP3*^Cre^*Bcl6*^fl/fl^ mice (n = 5-8/group) challenged with *B*. *melitensis* four weeks post challenge. **(B-D)** Quantification of the mean percentage of T_Reg_ (FoxP3^+^CXCR5^-^) **(B)**, T_FR_ (FoxP3^+^PD-1^+^CXCR5^+^) **(C)**, and T_FH_ (FoxP3^-^PD-1^+^CXCR5^+^) **(D)** of CD44 expressing CD4^+^ T cells in the spleen of infected animals four weeks post challenge. **(E)** Percentage of splenic GC B cells (Fas^+^GL7^+^ of CD19^+^) amongst CD19^+^ B cells four weeks post infection. **(F)** Splenic bacterial burdens of IgG or anti-CD20-treated *Bcl6*^fl/fl^ or *FoxP3*^Cre^*Bcl6*^fl/fl^ mice (n = 4-7/treatment) four weeks after challenge with *B*. *melitensis*. Data are representative of at least two independent experiments.

DTX treatment leads to T_FR_ deficiency in DEREG mice (S3J Fig) making it unclear whether T_Reg_, T_FR_ or both contribute to enhanced susceptibility to *Brucella*. Therefore, we employed FoxP3^Cre^*Bcl6*^fl/fl^ mice, in which T_FR_ are deficient while T_Reg,_ T_FH,_ and GC B cells remain intact [29] and found T_FR_ deficiency enhanced resistance to *Brucella* four weeks post infection (Fig 7A and 7F). FoxP3^Cre^*Bcl6*^fl/fl^ mice displayed elevated total T_Reg_ frequencies compared to *Bcl6*^*fl*/fl^ mice (Fig 7B), though this did not adversely affect control of infection. To determine whether T_FR_ require B cells to enhance susceptibility to infection, we depleted B cells from FoxP3^Cre^*Bcl6*^fl/fl^ and *Bcl6*^fl/fl^ mice. While isotype treated FoxP3^Cre^*Bcl6*^fl/fl^ mice were more resistant to infection than isotype treated *Bcl6*^fl/fl^ animals, *Brucella* burdens four weeks post infection were similar in FoxP3^Cre^*Bcl6*^fl/fl^ and *Bcl6*^fl/fl^ mice depleted of B cells (Fig 7F). Thus, our results demonstrate that, in the absence of B cells, T_FR_ are no longer deleterious to control of infection.

As T_FR_ regulate antibody responses [28,62,63], we questioned whether T_FR_ deficiency altered antibody responses which could in turn impact control of *Brucella*. While we did not find a difference in the quantity of *Brucella-*specific IgM generated by *Bcl6*^fl/fl^ and FoxP3^Cre^*Bcl6*^fl/fl^ mice four weeks post infection, FoxP3^Cre^*Bcl6*^fl/fl^ animals did display reduced anti-*Brucella* IgG levels (S4A Fig). However, passive transfer of sera from either *Bcl6*^*fl*/fl^ or FoxP3^Cre^*Bcl6*^fl/fl^ mice previously infected with *B*. *melitensis* conferred similar levels of protection to FoxP3^Cre^*Bcl6*^fl/fl^ mice (S4B Fig). Coupled with our finding that control of infection is not altered in mice lacking the ability to secrete antibody (Fig 1D), these data indicate the deleterious effect of T_FR_ is independent of antibody regulatory function.

## Discussion

We previously reported B cell mediated susceptibility to *Brucella* is CD4^+^ T cell dependent [19]. Here we report that an inability of B cells to recognize *Brucella* via BCR specificity results in host resistance to infection and reduced B cell uptake of *Brucella in vivo*. While B cells enhance expression of FoxP3 and decrease expression of CD44 and T-bet by CD4^+^ T cells in the first two weeks after infection (Figs 3A–3C and S1G), we found expression of CD44, T-bet, and FoxP3 was similar in CD4^+^ T cells from WT and MD4 mice at one- and two-weeks post-infection (S5A–S5F Fig). Total B cell deficiency results in a ~100-fold decrease in CFUs at 4 weeks post-infection (Figs 4B and 7F), while *Brucella* counts were reduced less than 10-fold in MD4 mice at the same timepoint (Fig 1A). The difference in phenotypes observed in MD4 mice versus mice with a total B cell deficiency could be explained by several factors. While ~90% of B cells in MD4 mice express a BCR specific for HEL [36], we did detect some *Brucella*-specific Ig MD4 mice (S1B and S1C Fig). Therefore, this residual population of non-HEL specific B cells in MD4 animals could potentially recognize *Brucella* through their BCR resulting in the dampened phenotype observed. Alternatively, while less efficient than antigen specific mechanisms, *Brucella* uptake and antigen presentation by B cells could also occur via BCR-independent processes [35].

Others have reported opsonization of *B*. *abortus* with IgM improves B cell uptake of bacteria *in vitro* [4]. However, we found the presence of *Brucella* specific antibody *in vivo* did not alter resistance to infection or reduce B cell *Brucella* burden (Fig 1D–1F). Interestingly, at later timepoints we found greater numbers of *Brucella* in B cells from *sIgM*^*-/-*^*/AID*^*-/-*^ mice (Fig 1E and 1F). B cells from mice that lack the ability to secrete IgM can have increased BCR signaling including elevated Btk activation [64]. This was of interest, as uptake of *Brucella* by B cells appears to be BCR specific (Fig 1), Btk is required for BCR-mediated antigen internalization [39], and because we found Btk deficiency enhances resistance to *Brucella* (Fig 2). Therefore, it is possible that enhanced BCR signaling in *sIgM*^*-/-*^*/AID*^*-/-*^ mice leads to increased uptake of *Brucella* by B cells. Alternatively, we found both the proportion and absolute number of B-1 cells in *sIgM*^-/-^/*AID*^-/-^ mice was increased >2.5 fold (S6A–S6H Fig), which is similar to what others have reported in *sIgM*^-/-^/*AID*^-/-^ mice [38]. As some B-1 cells can have phagocytic activity [65], the increased number of B-1 cells in *sIgM*^-/-^/*AID*^-/-^ mice could potentially lead to higher levels of *Brucella* within the B cell compartment.

*Brucella* burdens in the spleens of mice lacking B cell MHCII expression are ~10 fold lower than in control animals (Fig 2B), which is a lesser phenotype than the ~100-fold decrease in *Brucella* in mice depleted of B cells (Fig 7F) or in mice with a genetic B cell deficiency (Fig 4B). In the first week after infection, FoxP3 expression is lower while T-bet expression is higher on activated CD4^+^ T cells in μMT mice (Fig 3B and 3C); however, we found expression of FoxP3 and T-bet was similar in activated CD4^+^ T cells from B cell MHCII deficient mice and control animals one week post-infection (S7A–S7C Fig). In a previous study, we found increased levels of IFN-γ, TNF-α, IL-17, and IL-1β in the spleens of μMT mice at two weeks post-infection [19]. However, the levels of IFN-γ, TNF-α, IL-17, and IL-1β were similar in spleens of control and B cell MHCII deficient mice two weeks post-infection (S7D–S7I Fig). While CD19^Cre^ deletes the majority of MHCII expression on B cells in CD19^Cre^*iAB*^fl/fl^ mice, a fraction of B cells retains B cell MHCII expression, and the proportion of MHCII^+^ B cells in CD19^Cre^*iAB*^fl/fl^ mice increases over the course of infection from ~7% to ~25% (S8A Fig). Moreover, we observed a correlation between B cell MHCII expression and susceptibility to infection in CD19^Cre^*iAB*^fl/fl^ mice treated with IgG, but not in CD19^Cre^*iAB*^fl/fl^ mice depleted of CD4^+^ T cells (S8B and S8C Fig). Therefore, the potential/capacity for residual B cell MHCII expression and subsequent CD4^+^ T cell interaction following *Brucella* challenge in CD19^Cre^*iAB*^fl/fl^ mice could contribute to the diminished magnitude of the phenotype observed in these animals relative to total B cell deficiency.

Cotransfer of Fo B with CD4^+^ T cells inhibited the ability of CD4^+^ T cells to protect against infection (Fig 3F). Following initial antigen encounter, Fo B and T lymphocytes that have migrated to the B:T border reciprocally regulate the activation and differentiation of one another. This process relies on both B cell mediated antigen presentation and CD40:CD40L engagement [55]. Strikingly, CD40L blockade conferred protection against *Brucella* infection in WT, but not B cell deficient animals (Fig 4B). Similar to other reports [66], CD40L blockade suppressed T_FH,_ T_FR_ and GC B cell responses, signaling the GC response was ablated in treated animals (Figs 4C–4H and S2B–S2E). This suggests CD40L blockade hinders CD4^+^ T and Fo B CD40L:CD40 engagement during *Brucella* infection. Deletion of *Bcl6* in CD4^+^ T cells and CD40L blockade both thwart development of GC responses in *Brucella* infected mice yet have opposing effects on control of infection (Figs 4 and 5). CD40L blockade results in a decrease in the proportion of T_Reg_ (Fig 4F), while we observed that deficiency of *Bcl6* in CD4^+^ T cells results in an increase in the proportion and absolute number of T_Reg_ following *Brucella* infection (Figs 5H and S2H). Given that T_FR_ derive from thymic and/or peripheral T_Reg_ [22,24,26,67], the increase in T_Reg_ in CD4^Cre^*Bcl6*^fl/f^ mice during *Brucella* infection may arise from the inability of peripheral T_Reg_ to commit to T_FR_ differentiation in response to infection. *Bcl6* restrains CD4^+^ T cell IL-10 production [52], and T_Reg,_ along with other CD4^+^ T cell subsets, can produce IL-10 [68] which in turn promotes susceptibility of *Brucella* [69]. In particular, CD4^+^ T cell derived IL-10 diminishes TNF-α levels during *Brucella* infection [69], and we found *Bcl6* deficiency in CD4^+^ T cells markedly reduced TNF-α levels in the spleens of *Brucella*-infected mice (S9 Fig). Therefore, it is possible that an outgrowth of T_Reg_ and/or enhanced CD4^+^ T cell IL-10 production in CD4^Cre^*Bcl6*^fl/fl^ mice results in elevated susceptibility to *Brucella*, though this would require additional investigation to confirm.

Upon depletion of B cells from CD4^Cre^*Bcl6*^fl/f^ mice we observed a ~100-fold decrease in splenic bacterial burdens despite the absence of GC B cells in these animals (Fig 5B, 5C and 5I). This is of interest as it suggests GC B cells are not absolutely required for B cells to mediate enhanced susceptibility and implicates early CD4^+^ T and Fo B interactions as determinants of enhanced susceptibility to infection rather than interactions between fully committed T_FH_ and GC B cells in the GC.

PD-1 has been studied extensively in the context of chronic infection, particularly regarding its role in T cell exhaustion [57]. T cell responses to *Brucella* are inefficient at controlling infection, and CD8^+^ T cell exhaustion is associated with chronic disease in a murine model of brucellosis [11]. Intriguingly, we found PD-1 blockade enhanced susceptibility to infection in a CD4^+^ T and B cell-dependent manner (Fig 6A–6C). PD-1 restrains T_Reg_ and T_FR_ function [27,53,54] and upregulation of PD-1 by T_FH_ and T_FR_ contributes to follicular migration and positioning in the developing GC response [70]. Here PD-1 blockade enhanced susceptibility to *Brucella* and augmented T_Reg,_ and T_FR_ populations (Fig 6F and 6G). Notably, tumor infiltrating T_FR_ are prevalent in several types of cancer, and PD-1 blockade enhances tumor-infiltrating T_FR_ leading to reduced tumor control in mice [54]. Collectively, these findings support a link between PD-1 blockade and promotion of T_Reg_ and T_FR_ which may directly impact host susceptibility to infection and the efficiency of tumor control.

Alterations in T_Reg_ and T_FR_ frequencies induced by PD-1 blockade were B cell dependent (Fig 6F and 6G). B cell expression of PD-L1 is implicated in T_FH_ differentiation and migration [71], and PD-L1 expression by B cells has been linked to B cell regulatory function which can inhibit CD4^+^ T cell responses [72]. Interestingly, antigen-specific B cells protect against *Mycobacterium tuberculosis* infection, specifically through PD-L1 expressing B cells engaging PD-1^+^ T_FH_-like cells in the lung [73]. Future studies should focus on how B cell PD-L1/L2 expression affects the quantity and quality of T_FH,_ T_FR_ and T_Reg_ development during infection with *Brucella* and other pathogens.

While T_Reg_ and T_FR_ promote susceptibility to *Brucella*, only T_FR_ absolutely require B cells for this effect (Figs 7A and 7F and S3H). To gain insight as to how T_FR_ impact Fo B behavior to potentially alter susceptibility to infection, we performed RNA-seq on splenic Fo B isolated from *Bcl6*^fl/fl^ and FoxP3^Cre^*Bcl6*^fl/fl^ mice at two weeks post challenge, when *Brucella* burdens are similar in these strains (S9A Fig), and on Fo B from naïve animals (S10 Fig and S1 Table). When comparing the transcriptional profile of Fo B from infected *Bcl6*^fl/fl^ and FoxP3^Cre^*Bcl6*^fl/fl^ mice, 401 genes were differentially regulated. Interestingly, only ~5% of the genes differentially regulated when comparing Fo B obtained from infected *Bcl6*^fl/fl^ and FoxP3^Cre^*Bcl6*^fl/f^ mice were also differentially regulated when comparing Fo B from naïve *Bcl6*^fl/fl^ and FoxP3^Cre^*Bcl6*^fl/f^ mice (S10B and S10C Fig) indicating the effect of T_FR_ on Fo B transcription is context dependent. Interestingly, RNA-seq analysis revealed decreased Fo B transcription of *Tgfb3* in *Brucella*-infected T_FR_ deficient mice (S10A Fig). As TGF-β3 production by B cells has been shown to drive expansion of T_Reg_ [74], which are deleterious to control of *Brucella* (S3 Fig), regulation of B cell TGF-β3 expression by T_FR_ could alter susceptibility to *Brucella*. We also observed decreased transcription of dual specificity phosphatase 4 (*Dusp4*) in T_FR_ deficient mice (S10A Fig). *Dusp4* is induced upon B cell activation involving both CD40 engagement and BCR signaling and promotes apoptosis via negative regulation of JNK (1/2) [75]. As CD40:CD40L interactions and BCR signaling both play a part in B cell mediated susceptibility to *Brucella* (Figs 1 and 4), the role of alteration of *Dusp4* expression in B cells by T_FR_ may warrant further study. T_FR_ deficiency also altered transcription of genes involved in splenic compartmentalization/follicular positioning (*Klf2*, *Bcl6*, *Ccr6*, *Cxcr4*, *Pdlim1*) [70,71, 76–78], and antibody production (*Tgfβ3*) [79] which aligns with the established role of T_FR_ in conditioning B cell reactions during GC responses. Changes in the positioning of Fo B could alter interactions with CD4^+^ T cells. Thus, as B cell mediated susceptibility to *Brucella* is CD4^+^ T cell dependent ([19] and Fig 2), in the future we will investigate whether T_FR_ alter the positioning of Fo B and the frequency and/or magnitude of interactions of Fo B with CD4^+^ T cells.

Alternatively, T_FR_ may negatively impact the ability of non-B cell populations to effectively control infection. Due to the reciprocal regulation inherent in generation of T_FH_, T_FR_ and GC B responses [20,32,52], B cell deficiency lowers T_FR_ proportions (Fig 4E and 4H). Therefore, B cell dependent effects of T_FR_ may arise from their dependence on B cells for generation or maintenance. In this case, T_FR_ could either act directly on B cells, or could function to alter other T- and/or non-B cell populations. Interestingly, T_FR_ deficiency in mice has been linked to upregulation of granzyme B and other cytotoxicity associated genes in T_FH_ [33], indicating that T_FR_ can modulate the function of other CD4^+^ T cell populations.

In sum, our findings indicate B cells promote T_FR_ responses that are regulated by CD40L and PD-1 dependent mechanisms. T_FR_ in turn promote susceptibility to infection in a manner independent of the humoral response (S11 Fig). T_FR_ deficiency can enhance protection against influenza [62], but to our knowledge, this is the first report of an antibody independent effect of T_FR_ altering resistance to infection. Future studies will need to determine whether T_FR_ mediate B cell responses which in turn promote infection, or whether their deleterious effect is mediated by altering responses of non-B cell populations. Finally, investigating how B cell antigen specificity, presentation and T_FR_ induction synergize to hamper control of infection independent of the antibody response could have broad implications for rational vaccine designs which seek to optimize T_FH_ and GC B responses.

## Materials and methods

### Ethics statement

All mouse experiments were approved by the University of Missouri Animal Care and Use Committee (ACUC protocol 27761).

### Growth conditions and bacterial strains

All experiments were performed using *Brucella melitensis* 16M obtained from Montana State University (Bozeman, MT) in biosafety level 3 (BSL-3) facilities. Bacteria were grown on *Brucella* agar (Becton Dickinson) at 37°C/5% CO_2_ before colonies were picked and cultured in *Brucella* broth overnight at 37°C in an orbital shaker. Challenge doses were approximated by measurement of optical density at 600 nm and diluted using sterile Dulbecco’s Phosphate Buffered Saline (DPBS) (Thermofisher). All *in vivo* studies employed an intraperitoneal injection of 1x10^5^ CFUs of *B*. *melitensis* 16M in 200 μl of DPBS. The delivered dose was confirmed via plating of inoculum onto *Brucella* agar.

### Mice

Animals challenged with *B*. *melitensis* were of mixed sex and were age (6–12 weeks) and sex matched for all experiments. Mice were maintained in individually ventilated caging under high efficiency particulate air-filtered barrier conditions with 12 hr light and dark cycles within ABSL-3 facilities at the University of Missouri. Food and water were provided to animals ad libitum. B6.129S2-*Ighm*^*tm1Cgn*^/J (μMT), B6.129S7-Rag1tm1Mom/J (*Rag1*^-/-^), C57BL/6-Tg(IghelMD4)4Ccg/J (MD4), B6.129X1-*H2-Ab1*^*tm1Koni*^/J (*iAB*^*fl/fl*^), C.B6-Tg(Foxp3-DTR/EGFP)23.2Spar/Mmjax (DEREG), B6.129S(FVB)-Bcl6tm1.1Dent/J (*Bcl6*^fl/fl^), CBA/CaJ, and CBA/CaHN-Btk^XID^/J (XID) and C57BL/6J (WT) mice were obtained from the Jackson Laboratory. With the exception of XID mice and their controls (CBA/CaJ mice), all animal strains were on a C57BL/6 background. *sIgM*^-/-^/*AID*^-/-^ mice were a gift from Dr. Nicole Baumgarth at the University of California, Davis. *AID*^-/-^ mice were originally generated at Kyoto University [80] and were bred to *sIgM*^-/-^ mice at the Trudeau Institute [81]. B6.129P2(C)-Cd19tm1(cre)Cgn/J (CD19^Cre^) and B6.Cg-Tg(Cd4-cre)1Cwi/BfluJmice (CD4^Cre^) were a gift from Dr. Mark Daniels (University of Missouri). CD19^Cre^ animals were intercrossed with *iAB*^fl/fl^ animals to generate CD19^Cre^*iAB*^fl/fl^ mice. *FoxP3*^Cre+/+^*Bcl6*^fl/fl^ (Bcl6FC) animals were gifted from Dr. Alexander Dent (University of Indiana). CD4^Cre^ and *FoxP3*^Cre+/+^ animals were intercrossed with *Bcl6*^fl/fl^ mice to generate *Cd4*^Cre^*Bcl6*^fl/fl^ and *FoxP3*^Cre+/+^*Bcl6*^fl/fl^ respectively. Experiments involving challenge of MD4, or CD19^Cre^*iAB*^fl/fl^ mice utilized HEL-negative or *iAB*^fl/fl^ litter mates as control animals respectively. In all experiments using CD4^Cre^*Bcl6*^fl/fl^ or *FoxP3*^*Cre*^*Bcl6*^fl/fl^ animals, *Bcl6*^fl/fl^ mice were used as controls. A description of the phenotypes of mice employed in this study are shown in Table 1.

**Table 1:** Mouse Strains used in this Study.

Mouse Strain	Phenotype
C57BL/6	Wild-type (WT) mice
MD4	Express BCR specific for Hen Egg Lysozyme
*sIgM* ^*-/-*^ */AID* ^*-/-*^	Cannot secrete IgM or class switched antibodies
XID	Defect in Bruton’s tyrosine kinase
CBA/J	Control for XID mice
CD19^Cre^*iAB*^fl/fl^	Lack B cell MHCII expression
*iAB* ^fl/fl^	Control for CD19^Cre^*iAB*^fl/fl^ mice
μMT	Lack B cells
CD4^cre^*Bcl6*^fl/fl^	Lack T follicular helper cells
FoxP3^cre^*Bcl6*^fl/fl^	Lack T follicular regulatory cells
CD4^cre^*Bcl6*^fl/fl^	Control for CD4^cre^*Bcl6*^fl/fl^ and FoxP3^cre^*Bcl6*^fl/fl^ mice
DEREG	Express diphtheria toxin receptor under control of FoxP3 promoter
*Rag1* ^-/-^	Lack T and B cells

### Quantification of bacterial burden

Spleens were mechanically homogenized, serially diluted, and aliquots plated in triplicate onto *Brucella* agar as previously described [82]. Plated samples were incubated for 3–4 days at 37°C/5%CO_2_, and colonies counted to quantify the total CFUs/tissue. For enumeration of viable intracellular B cell burdens, B cells were isolated from spleens of infected animals using positive selection with anti-CD19 magnetic bead isolation (Miltenyi Biotec). For each sample, an aliquot of spleen homogenate was used for B cell purification, and total splenic cells, total B cells harvested, and total B cells/spleen were calculated. To kill extracellular bacteria, spleen homogenates were incubated in complete medium (CM; RPMI 1640, 0.1 HEPES, 1 mM sodium pyruvate, 1 mM nonessential amino acids, and 10% fetal bovine serum [FBS]) containing 50 μg/ml gentamicin for 30 minutes. Homogenates were then washed, and the remaining isolation protocol carried out using MACs isolation buffer (PBS, pH 7.2, 0.5% BSA, and 2 mM EDTA) supplemented with 5 μg/mL gentamicin. CD19^+^ and CD19^-^ fractions were washed three times with DPBS and lysed in icy cold molecular grade water. Cell lysates were plated in triplicate to determine the *Brucella* burden for each fraction. Flow cytometric analysis of an aliquot of each sample was performed to confirm CD19^+^ B cell fractions were >90% pure. For measurement of cytokines, homogenized tissues were centrifuged at 2000 X G for 5 minutes, and supernatants were filter sterilized (0.22 μm) and stored at −70°C prior to analysis. Cytokines were measured with a Luminex (Austin, TX) MagPix instrument using Milliplex magnetic reagents according to manufacturer’s instructions (MilliporeSigma, Burlington, MA). Luminex data were analyzed with Milliplex Analyst Software (MilliporeSigma).

### Adoptive transfers

Spleens were collected from naïve animals and mechanically homogenized. For CD4^+^ T cell and total B cell isolations, cells were magnetically purified using either CD4 or CD19 magnetic bead isolation kits (Miltenyi Biotec). Fo B cells were isolated from spleen using the MZ and FO B cell isolation kit (Miltenyi Biotec). Isolated lymphocytes were transferred via intravenous injection in 200 μL of DPBS into the tail vein of recipient mice one day prior to challenge with *B*. *melitensis*. Each animal received ~1.5 x 10^7^ CD4^+^ T cells alone, ~1.5 x 10^7^ CD4^+^ T cells in tandem with 5 x 10^7^ (total B cell), or ~1.5 x 10^7^ CD4^+^ T cells in tandem with 3-5x10^7^ Fo B cells. For B-1a cell transfers, cells were harvested from the peritoneal cavity and pleural space of naïve mice [83]. Cells from the peritoneum and pleural cavity were pooled and B-1a cells purified using the mouse B-1a cell isolation kit (Miltenyi Biotec). B-1a transfer groups received ~2x10^5^ B-1a cells in 200 μl of PBS or ~2x10^5^ B-1a cells in 200 μl of PBS concomitant with intravenous administration of CD4^+^ T cells (1.5x10^7^). The purity of sorted cell populations was confirmed to be ≥90% purity via flow cytometry. Purified CD4^+^ T and total B cells were assessed using anti-CD4 (GK1.5 Biolegend), anti-CD3 (145–2011 Biolegend), anti-CD8 (53–6.7 eBioscience), anti-CD19 (1D3 Leinco) and/or anti-B220 (RA3-6B2 Biolegend). B-1a cell purity was determined using anti-CD19 (1D3 Leinco) and/or anti-B220 (RA3-6B2 Biolegend), anti-CD5 (53–7.3 Biolegend), and anti-CD43 (S11 Biolegend).

### Passive antibody transfer

*Bcl6*^*fl/fl*^ and *FoxP3*^Cre^*Bcl6*^fl/fl^ mice were challenged with *B*. *melitensis* and whole blood drawn via intracardial exsanguination four weeks post infection. Sera were collected by centrifuging blood samples at 10,000X G for 10 minutes at room temperature. Sera samples were stored at -80°C until passive transfer. Anti-*Brucella* IgM and IgG were quantified via ELISA as described below. For passive transfer, sera from each individual *Bcl6*^fl/fl^ or *FoxP3*^Cre^*Bcl6*^fl/fl^ sample were sterilized using a 0.22 μm filter and pooled by genotype. 200 μL from either *Bcl6*^fl/fl^ or *FoxP3*^Cre^*Bcl6*^fl/fl^ pooled stock were administered i.p. to naïve *FoxP3*^Cre^*Bcl6*^fl/fl^ mice twenty-four hours prior to infection.

### Anti-*Brucella* antibody ELISA

ELISA performed as previously described with minor modifications [8,84]. Briefly, 96-well high binding plates (Nunc), were coated overnight at 4°C with 10^8^ CFU equivalents of the heat killed *B*. *abortus* S19 vaccine strain (University of Wyoming) in 0.05 M carbonate/bicarbonate coating buffer (pH 9.6). For measuring total Ig levels and for the standard curve, either unlabeled rat anti-mouse IgM (5 μg/ml) or goat anti-mouse IgG (0.5 μg/ml) (Southern Biotech) were used to coat IgM or IgG ELISA plates respectively. Plates were then washed using PBS-T buffer (0.05% Tween-20 in 1x PBS) before blocking for 1 hr at room temperature using 1% BSA in PBS. Plates were washed again before addition of serially diluted serum, or serially diluted IgM/IgG (standard curve) and allowed to incubate for 2 hrs at room temperature. ELISA plates were subsequently washed, and Goat anti-mouse IgM-HRP (1:1000) or Goat anti-mouse IgG-HRP (1:4000) antibody (Southern Biotech) added before incubation for 1 hr at room temperature. Plates were washed a final time before development with TMB substrate (Invitrogen) and addition of stop solution (2N Sulfuric acid solution). Absorbance was measured at 450 nm using a SpectraMax (Molecular Devices, San Jose, CA). Standard curves with unlabeled mouse IgM or IgG were employed to estimate Ig concentrations and data are presented in Units/ml (U/ml), where 1 U/ml roughly correlates with 1 pg/ml of antibody. The limit of detection for anti-*Brucella* antibody was 30.9 U/ml for IgM, and 3.43 U/ml for IgG.

### Flow cytometry

Spleens were homogenized and cell suspensions filtered through sterile 40 μm mesh following red blood cell lysis. Splenocytes were Fc blocked (2.4G2 Leinco) in fluorescence-activated cell-sorting (FACS) buffer (2% heat inactivated fetal bovine serum in DPBS) before extracellular staining with fluorochrome-conjugated mAbs: anti-CD4 (GK1.5 Biolegend), anti-CXCR5 (L138D7 Biolegend), anti-CD44 (IM7 Biolegend), anti-CD23 (B3B4 Biolegend), anti-CD19 (1D3 Biolegend) or anti-CD19 (6D5 Biolegend), anti-CD43 (S11 Biolegend), anti-CD21 (7E9 Biolegend), anti-B220 (RA3-6B2 eBioscience), anti-CD8 (53–6.7 Biolegend), anti-CD3 (145-2C11 Biolegend or BD Biosciences), anti-I-A/I-E(MHCII) (M5/114.15.2 Biolegend), anti-CD279 (PD-1) (29F.1A12 Biolegend), anti-CD278 (ICOS) (7E.17G9 BD Biosciences), anti-mu/HU GL7 antigen (GL7 Biolegend), anti-CD95 (Fas) (SA367H8 Biolegend), and eBioscience Fixable Viability Dye eFluor 780 (Invitrogen). Cells were then fixed in 4% formalin at 4°C overnight before washing with and resuspension in FACS buffer. For intracellular staining, samples were fixed and permeabilized using the eBioscience Foxp3/Transcription Factor Staining Buffer Set (Thermofisher) for 30 minutes at room temperature following extracellular staining. Samples were then stained with anti-T-bet (4B10 Biolegend) and anti-FoxP3 (FJK.16s Invitrogen) for two hours before fixation with 4% formalin at 4°C overnight, washing and resuspension in FACS buffer. Fluorescence was measured using a CyAn ADP High-Performance Flow Cytometer, BD LSR Fortessa X-20, or a Cytek Aurora spectral analyzer. Fluorescence Minus One (FMO) controls were utilized to identify rare or dim populations such as CXCR5 and PD-1 expressing cells (S12 Fig). Data were analyzed using FlowJo (Tree Star) software.

### *In vivo* cell depletions

Animals were depleted of CD4^+^ or CD8^+^ T cells as previously described [19,85]. Briefly, animals were treated with 0.5 mg of rat anti-CD4 mAb GK1.5 (Leinco) or 0.2 mg rat anti-CD8 mAb 2.43 (Leinco) in 200 μl of DPBS i.p. one day prior to challenge. Treatment was repeated once weekly for the entirety of each study. For depletion of B cells, animals were treated with 250 μg of rat anti-CD20 mAB MB20-11 (BioXcell) in 200μl of DPBS i.p. one week prior to challenge [86]. Anti-CD20 treatment was repeated on day 14 post infection. Control animals for both T and B cell depletions received equivalent dosages of rat or mouse IgG (Leinco or Southern Biotech) respectively. Upon euthanasia, depletion of splenic CD4^+^ T, CD8^+^ T, or B cell populations were confirmed to be ≥ 90% effective via flow cytometry. T_Reg_ were depleted in DEREG animals by administering 1 μg DTX (List Biological Laboratories) resuspended in 100 μL DPBS i.p. to each animal on day 14 and 15 post challenge [59]. WT animals were administered an identical dose of DTX as a control. Blood was collected from animals to confirm systemic depletion of FoxP3^+^CD4^+^ T cells seven days post treatment (S3A Fig).

### CD40L and PD-1 blockade

WT and/or μMT animals were administered 250 μg, hamster anti-CD154 (CD40L) mAB MR-1 (BioXcell), or 250 μg of rat anti-CD279 (PD-1) mAB RMP1-14 (Leinco) in 200 μL of DPBS i.p. one day prior to the start of the study. Treatments were repeated every three days thereafter for the duration of the study based on the regimens of others [87,88]. Control animals were treated with equivalent dosages of either hamster IgG (CD40L) (Southern Biotech) or rat IgG (PD-1) (Leinco).

### RNA-Seq analysis

Fo B cells were sorted from naïve or infected animals as described above, and were washed, and placed in 1 ml of RNAlater (Thermo) and stored at 4°C overnight. The B cell purity of cells isolated from *Bcl6* and *FoxP3*^Cre^*Bcl6*^fl/fl^ averaged 93.5% and 97.4% respectively. Fo B cell (CD23^+^CD21^lo^B220^+^) purity averaged ~85% of live B220^+^ cells as determined by flow cytometry in both strains. Approximately 4x10^6^-1x10^7^ Fo B were harvested from each animal. RNA was purified according to manufacturer instructions using a RNeasy Mini kit (Qiagen). Poly A enriched stranded mRNA libraries were generated, which were then sequenced on a NovaSeq 6000 (Illumina) as described elsewhere [89]. RNA-seq data were analyzed by the University of Missouri Bioinformatics Core Facility. Initial quality control of raw paired-end reads (100bp) was performed FastQC (v.0.11.8, https://www.bioinformatics.babraham.ac.uk/projects/fastqc/). Subsequently, fastp [90] with default parameters was used to remove adapter sequences and quality trim reads. Trimmed reads were aligned to the mouse genome assembly (mm39, annotation V109, Ensembl: http://useast.ensembl.org/Mus_musculus/Info/Index) and gene read count was quantified using STAR [91]. The gene counts for each sample were transformed and normalized using the variance-stabilizing transformation method implemented in the Bioconductor package DESeq2 [92] in R (v4.2.1; https://www.r-project.org/). Linear regression models within DESeq2 were used to identify differentially expressed genes between case vs. control sample sets. Final values for differential expression are log2 fold change ≥ 1 or ≤ -1 with false discovery rate < 0.05 (FDR, Benjamini-Hochberg) as significant.

### Statistical analysis

All comparisons of means between two groups were assessed via Student *t* test with significance set at *P* ≤ 0.05. Comparisons of three or more groups were conducted using one-way ANOVA, followed by Tukey’s test for correction of multiple comparisons unless otherwise noted. For all experiments, error bars represent the standard deviation of the sample mean. N values and the number of experimental repeats are provided in the figure legends. All statistical analyses were performed with Prism software (version 9.2, GraphPad) and all error bars indicate standard deviation (S.D.). Statistically significant differences are indicated as *, P ≤ 0.05; **, P ≤ 0.01; ***, P ≤ 0.001; ****, P ≤ 0.0001; and NS, not significant.

## Supporting information

S1 Fig MD4 animals have reduced anti-*Brucella* antibody levels. Total IgM and IgG levels were measured in the serum of MD4 and WT mice (n = 8–10 group) four weeks after infection with *B*. *melitensis* **(A)**. Serum anti-*Brucella* IgM **(B)** and IgG **(C)** in MD4 and WT mice (n = 5-11/group/time point) at two- and four-weeks post challenge with *B*. *melitensis*. Total B cell (CD19^+^) numbers **(D)** were determined in the spleens of WT and MD4 mice (n = 8-10/group/time point) 1, 2, or 4 weeks after infection challenge with *B*. *melitensis*. **(E)** Splenic *Brucella* burdens four weeks post challenge of XID mice (n = 5/treatment) adoptively transferred B-1a cells or DPBS one day prior to infection. **(F-I)** Quantification of splenic CD4^+^ T cell responses assessed via flow cytometry in *B*. *melitensis* infected animals. T-bet **(F)** and FoxP3 **(G)** were measured on CD44^+^CD4^+^ T cells in WT and μMT mice (n = 3-6/group/time point) at two weeks post infection. FoxP3 **(H)** and T-bet **(I)** were assessed on CD44^+^CD4^+^ T cells in *Rag1*^-/-^ mice (n = 5-6/treatment) that received either CD4^+^ T cells alone, or CD4^+^ T cells in tandem with B cells, one day prior to challenge with *B*. *melitensis*. **(J)** Splenic bacterial burdens in *Rag1*^-/-^ mice (n = 5/treatment) administered PBS, CD4^+^ T cells alone, or CD4^+^ T cells in combination with B-1a cells one day prior to *B*. *melitensis* challenge. Data in **(A-C and E-I)** are from a single experiment. Data in **(D)** are pooled from two experiments, and data in **(J)** are representative of at least two independent experiments.
(TIF)

S2 Fig Enhanced susceptibility of CD4^Cre^*Bcl6*^fl/fl^ mice is CD8^+^ T cell independent. The total number of CD19^+^ B cells (A), germinal center B cells (CD19^+^Fas^+^GL7^+^) (B), T_Reg_ (FoxP3^+^CXCR5^-^CD44^+^CD4^+^) (C), T_FH_ (FoxP3^-^ICOS^+^CXCR5^+^CD44^+^CD4^+^) (D) and T_FR_ (FoxP3^+^ICOS^+^CXCR5^+^CD44^+^CD4^+^) (E) were counted in the spleens of WT mice treated with IgG or anti-CD40L (n = 5/treatment) four weeks after infection with *B*. *melitensis*. Splenic bacterial burdens (F) in *Bcl6*^fl/fl^ and CD4^Cre^*Bcl6*^fl/fl^ mice (n = 4-5/treatment) treated with CD8-depleting antibody or IgG as an isotype four weeks after *B*. *melitensis* infection. (G) Quantification of the percent T-bet^+^ cells amongst activated (CD44^+^) CD4^+^ T cells in the spleens of *Bcl6*^fl/fl^ and CD4^Cre^*Bcl6*^fl/fl^ animals (n = 4-5/group) four weeks post *B*. *melitensis* infection. The number of T_Reg_ (FoxP3^+^CXCR5^-^CD44^+^CD4^+^) (H) was determined four weeks after *B*. *melitensis* infection in the spleens of *Bcl6*^fl/fl^ and CD4^Cre^*Bcl6*^fl/fl^ mice (n = 5-6/treatment). Data in (A-F and H) are from a single experiment, and data in (G) are representative of at least two independent experiments.
(TIF)

S3 Fig T_Reg_ driven enhanced susceptibility to *Brucella* infection does not absolutely require B cells. WT or DEREG mice (n = 5/group) were challenged with *B*. *melitensis* and treated with DTX on D14 and D15 post infection. (A) Quantification of the percentage of T_Reg_ amongst CD44^+^CD4^+^ cells in the blood of WT and DEREG mice seven days post DTX treatment. (B) Splenic bacterial burdens in DTX-treated WT and DEREG animals. (C-D) Representative flow plots of the gating strategy (C) and quantification of (D) the percentage of B cells present in the spleens of DTX treated WT and DEREG animals four weeks post infection. (E-F) Representative flow plots (E) and quantification of the percentage of GC B cells amongst CD19^+^ B cells (F) present in the spleens of DTX-treated WT and DEREG mice. (G) Percentage of Th1 effector cells (T-bet^+^) amongst CD44^+^CD4^+^ T cells in WT and DEREG DTX-treated animals. (H) WT and DEREG mice (n = 6-8/treatment) treated with DTX and anti-CD20 or IgG isotype control and CFUs were measured four weeks post-infection. (I) Representative flow plots of the gating strategy to identify T_FR_ (FoxP3^+^CXCR5^+^PD-1^+^) and T_FH_ (FoxP3^-^CXCR5^+^PD-1^+^) within splenic tissue in infected animals. (J-K) Quantification of the indicated populations of T_FR_ (J) and T_FH_ (K) amongst CD44^+^CD4^+^ T cells in DTX treated WT and DEREG animals. Data in (H) are combined from two independent experiments. All other data are from a single experiment.
(TIF)

S4 Fig T_FR_ enhance susceptibility to *Brucella* independent of antibody. **(A)** Anti-*Brucella* IgM and IgG titers four weeks post *B*. *melitensis* challenge in the serum of *Bcl6*^fl/fl^ and *FoxP3*^Cre^*Bcl6*^fl/fl^ animals (n = 10/group). **(B)** Splenic bacterial burdens of *FoxP3*^Cre^*Bcl6*^fl/fl^ mice (n = 4-5/transfer) administered pooled sera from previously infected *Bcl6*^fl/fl^ or *FoxP3*^Cre^*Bcl6*^fl/fl^ mice one day prior to infection with *B*. *melitensis*. Data are from a single experiment.
(TIF)

S5 Fig CD4^+^ T cell responses in MD4 mice. MD4 mice or WT littermates (n = 3-5/group/timepoint) were challenged i.p. with 1x10^5^ CFUs of *B*. *melitensis* 16M. At one **(A-C)** or two **(D-F)** weeks post-infection flow cytometry was performed to assess CD44 expression on CD4^+^ T cells **(A,D)**, and the expression of T-bet **(B,E)** and FoxP3 **(C,F)** on CD44 expressing CD4^+^ T cells. Data are from a single experiment.
(TIF)

S6 Fig B cell populations in WT and *sIgM*^*-/-*^*/AID*^*-/-*^ mice. WT or *sIgM*^*-/-*^*/AID*^*-/-*^ mice (n = 4-5/group) were challenged i.p. with 1x10^5^ CFUs of *B*. *melitensis* 16M. At four weeks post-infection the proportion **(A-D)** and number **(E-H)** of CD19^+^ B cells **(A,E)**, Fo B cells (CD23^+^CD21^lo^CD43^-^CD19^+^) **(B,F)**, MZ B cells (CD23^lo^CD21^+^CD43^-^CD19^+^) **(C,G)**, and B-1 cells (CD43^+^CD19^+^) (**D,H)** was determined in the spleen. Data are from a single experiment.
(TIF)

S7 Fig CD4^+^ T cell responses and cytokine levels in mice lacking B cell MHCII expression. CD19^Cre^*iAB*^fl/fl^ or *iAB*^fl/fl^ littermates (n = 5-6/group) were challenged i.p. with 1x10^5^ CFUs of *B*. *melitensis* 16M (A-C). At one-week post-infection, flow cytometry was performed to assess CD44 expression on CD4^+^ T cells (A), and the expression of T-bet (B) and FoxP3 (C) on CD44 expressing CD4^+^ T cells. CD19^Cre^*iAB*^fl/fl^ or *iAB*^fl/fl^ littermates (n = 3-7/group) were challenged i.p. with 1x10^5^ CFUs of *B*. *melitensis* 16M (D-I). Two weeks after infection, colonization of the spleen was measured (D) and the splenic levels of IFN-γ (E), TNF-α (F), IL-17 (G), IL-1β (H) and IL-4 (I) were determined. Data are from a single experiment.
(TIF)

S8 Fig Residual MHCII expression on B cells from CD19^Cre^*iAB*^fl/fl^ mice. CD19^Cre^*iAB*^fl/fl^ or *iAB*^fl/fl^ littermates (n = 5-6/group) were challenged i.p. with 1x10^5^ CFUs of *B*. *melitensis* 16M **(A)**. At one and four weeks after challenge flow cytometry was performed to assess MHCII expression on CD19^+^ B cells **(A)**. CD19^Cre^*iAB*^fl/fl^ mice (n = 5/group) were treated with IgG **(B)** or anti-CD4 **(C)** and challenged i.p. with 1x10^5^ CFUs of *B*. *melitensis* 16M. Four weeks after infection, colonization of the spleen was plotted against B cell MHCII expression in these animals in order to perform a linear regression. Data are from a single experiment.
(TIF)

S9 Fig Cytokine levels in mice lacking T_FH_ or T_FR_. *Bcl6*^fl/fl^, CD4^Cre^*Bcl6*^fl/fl^ or FoxP3^Cre^*Bcl6*^fl/fl^ mice (n = 4-6/group) were challenged i.p. with 1x10^5^ CFUs of *B*. *melitensis* 16M **(A-F)**. Two weeks after infection, colonization of the spleen was measured **(A)** and the splenic levels of IFN-γ **(B)**, TNF-α **(C)**, IL-17 **(D)**, IL-1β (**E)** and IL-4 **(F)** were determined. Data are from a single experiment.
(TIF)

S10 Fig T_FR_ deficiency alters Fo B transcription during *Brucella* infection. RNA-Seq of Fo B (n = 3-5/group) from T_FR_ deficient and control animals. **(A)** Z-scored heat map of selected genes differentially expressed in Fo B amongst naive *Bcl6*^fl/fl^ and *FoxP3*^Cre^*Bcl6*^fl/fl^ and infected *Bcl6*^fl/fl^ and *FoxP3*^Cre^*Bcl6*^fl/fl^ mice 14 days post *Brucella* challenge. **(B)** Table depicting the total number of differentially expressed genes (DE genes) detected when comparing infected and naïve *Bcl6*^fl/fl^ and *FoxP3*^Cre^*Bcl6*^fl/fl^ animals (filtering criteria: FDR <0.05 and Log_2_ FC ≥ 1 or Log_2_ FC ≤ -1). **(C)** Venn diagram depicting the percent overlap of differentially expressed genes. Data are from a single experiment.
(TIF)

S11 Fig Working model. **(A)** Ag-specific B cell presentation to splenic CD4^+^ T cells results in inefficient CD4^+^ T cell mediated control of infection in WT mice. T_FR_, T_FH_, GC B and *Brucella*-specific antibody responses develop in response to infection. **(B)** Inhibition of Fo B and CD4^+^ T cell interaction in WT animals via treatment with CD40L blocking antibody results in enhanced control of splenic *Brucella* burdens. T_FR_, T_FH_ and GC B responses are suppressed, suggesting one or more of these populations may enhance susceptibility during infection. **(C)** Alteration of Fo B and CD4^+^ T cell regulation via PD-1 blockade promotes T_FR_ outgrowth and enhances susceptibility to *Brucella*. **(D)** Genetic T_FR_ specific deficiency results in reduced splenic *Brucella* loads despite similar T_FH_, GC B cell and *Brucella*-specific antibody responses compared to control animals. This indicates T_FR_ promote susceptibility during *Brucella* infection through a mechanism that is independent of their role in shaping the humoral response to infection. Image created with Biorender.
(PNG)

S12 Fig FMO controls. A representative plot including a sample, and fluorescence minus one (FMO) controls to show how PD-1 and CXCR5 expressing CD4^+^ T cell populations were identified.
(TIF)

S1 Table Table depicting expression of genes in Fo B isolated from *Brucella* infected and naïve *Bcl6*^fl/fl^ and *FoxP3*^Cre^*Bcl6*^fl/fl^ animals (related to S10 Fig). (XLSX)
