# Supplementary figures and images for: Interactions between B cells and T follicular regulatory cells enhance susceptibility to Brucella infection independent of the anti-Brucella humoral response
Source: PLoS Pathog. 2023 Sep 18;19(9):e1011672. doi: 10.1371/journal.ppat.1011672 (PMC10538787; doi:10.1371/journal.ppat.1011672)

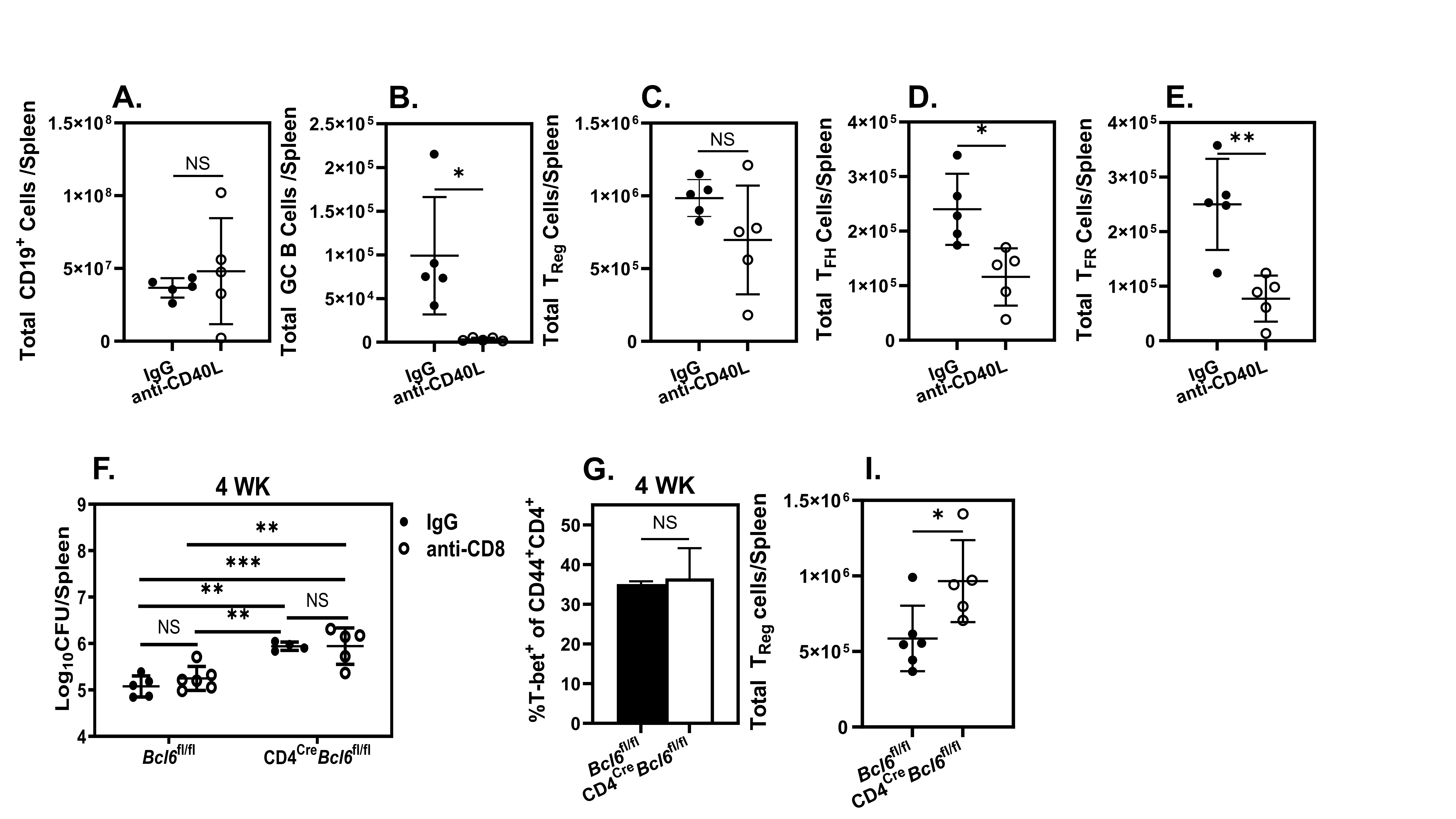

Supplement: S2 Fig — The total number of CD19+ B cells (A), germinal center B cells (CD19+Fas+GL7+) (B), TReg (FoxP3+CXCR5-CD44+CD4+) (C), TFH (FoxP3-ICOS+CXCR5+CD44+CD4+) (D) and TFR (FoxP3+ICOS+CXCR5+CD44+CD4+) (E) were counted in the spleens of WT mice treated with IgG or anti-CD40L (n = 5/treatment) four weeks after infection with B. melitensis. Splenic bacterial burdens (F) in Bcl6fl/fl and CD4CreBcl6fl/fl mice (n = 4-5/treatment) treated with CD8-depleting antibody or IgG as an isotype four weeks after B. melitensis infection. (G) Quantification of the percent T-bet+ cells amongst activated (CD44+) CD4+ T cells in the spleens of Bcl6fl/fl and CD4CreBcl6fl/fl animals (n = 4-5/group) four weeks post B. melitensis infection. The number of TReg (FoxP3+CXCR5-CD44+CD4+) (H) was determined four weeks after B. melitensis infection in the spleens of Bcl6fl/fl and CD4CreBcl6fl/fl mice (n = 5-6/treatment). Data in (A-F and H) are from a single experiment, and data in (G) are representative of at least two independent experiments. (TIF) [file ppat.1011672.s002.tif]

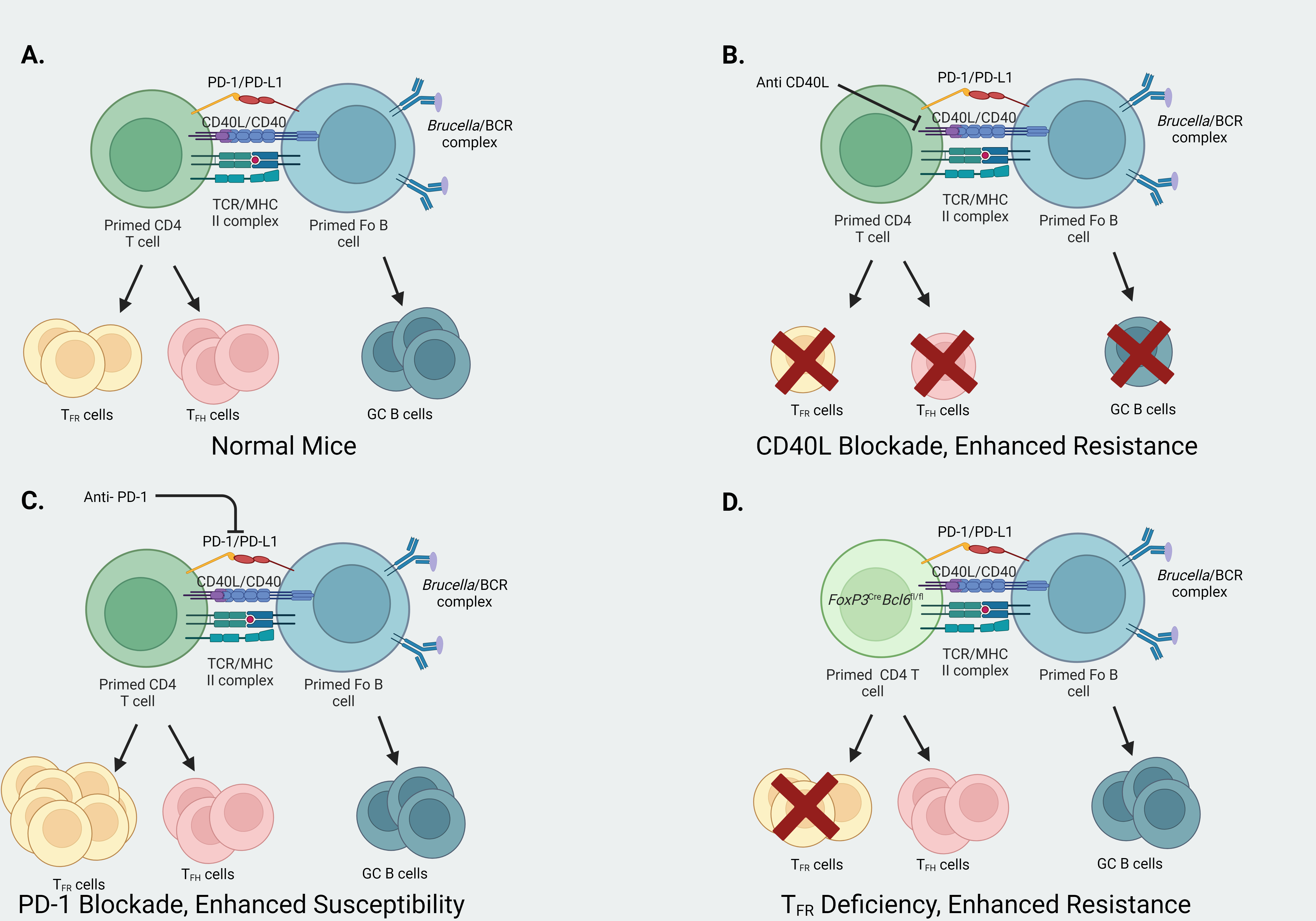

Supplement: S11 Fig — (A) Ag-specific B cell presentation to splenic CD4+ T cells results in inefficient CD4+ T cell mediated control of infection in WT mice. TFR, TFH, GC B and Brucella-specific antibody responses develop in response to infection. (B) Inhibition of Fo B and CD4+ T cell interaction in WT animals via treatment with CD40L blocking antibody results in enhanced control of splenic Brucella burdens. TFR, TFH and GC B responses are suppressed, suggesting one or more of these populations may enhance susceptibility during infection. (C) Alteration of Fo B and CD4+ T cell regulation via PD-1 blockade promotes TFR outgrowth and enhances susceptibility to Brucella. (D) Genetic TFR specific deficiency results in reduced splenic Brucella loads despite similar TFH, GC B cell and Brucella-specific antibody responses compared to control animals. This indicates TFR promote susceptibility during Brucella infection through a mechanism that is independent of their role in shaping the humoral response to infection. Image created with Biorender. (PNG) [file ppat.1011672.s011.png]
